# Wildfires and the role of their drivers are changing over time in a large rural area of west-central Spain

**DOI:** 10.1038/s41598-018-36134-4

**Published:** 2018-12-12

**Authors:** O. Viedma, I. R. Urbieta, J. M. Moreno

**Affiliations:** Departamento de Ciencias Ambientales, Universidad de Castilla-La Mancha, Avda. Carlos III, 45071 Toledo, Spain

## Abstract

During the last decades, wildfires have been changing in many areas across the world, due to changes in climate, landscapes and socioeconomic drivers. However, how the role of these drivers changed over time has been little explored. Here, we assessed, in a spatially and temporally explicit way, the changing role of biophysical and human-related factors on wildfires in a rural area in west-central Spain from 1979 to 2008. Longitudinal Negative Binomial (NB) and Zero-Inflated Negative Binomial (ZINB) mixed models, with time as interacting factor (continuous and categorical), were used to model the number of fires of increasing size (≥1–10 ha, >10–100 ha, >100 ha) per 10 × 10 km cell per year, based on fire statistics. We found that the landscape was rather dynamic, and generally became more hazardous over time. Small fires increased and spread over the landscape with time, with medium and large fires being stable or decreasing. NB models were best for modelling small fires, while ZINB for medium and large; models including time as a categorical factor performed the best. Best models were associated to topography, land-use/land cover (LULC) types and the changes they underwent, as well as agrarian characteristics. Climate variables, forest interfaces, and other socioeconomic variables played a minor role. Wildfires were initially more frequent in rugged topography, conifer forests, shrublands and cells undergoing changes in LULC types of hazardous nature, for all fire sizes. As time went by, wildfires lost the links with the initial fire-prone areas, and as they spread, became more associated to lower elevation areas, with higher solar radiation, herbaceous crops, and large size farms. Thus, the role of the fire drivers changed over time; some decreased their explaining power, while others increased. These changes with time in the total number of fires, in their spatial pattern and in the controlling drivers limit the ability to predict future fires.

## Introduction

During the last decades, wildfires have been changing in many areas across the world. While changes in climate are often the main factors^1–3^, changes in landscapes and socioeconomic factors are also associated to such variations^4–6^. That is particularly important in areas where humans are the major source of ignitions and of landscape change and its flammability. Hence, addressing how human factors have affected wildfires in recent times is important and challenging, due to its varying nature, together with continuing changes in other fire drivers.

At regional and local scales, human factors play a major role in wildfires, overriding in part the role of climate^7,8^. Human factors exert a dual effect on fire regime, by either decreasing fires (e.g., suppression policy^9^) or increasing them (e.g., Land Use/Land Cover [LULC] changes^10^), and can dampen fire-climate relationship at different spatial and temporal scales^8^. In the European Mediterranean countries (EUMed), the collapse of traditional rural socioeconomic systems since the second half of the XXth century have caused important landscape and socioeconomic changes, increasing landscape flammability and fire risk^10,11^. Specifically, fire risk has increased at both the wildland-urban interfaces (WUIs) and wildland-agrarian interfaces (WAIs), where population and human infrastructures are in contact with forest areas and there is an intense competition between agricultural and forestry activities^12–14^.

Nonetheless, the effects of those driving factors on wildfires vary across temporal and spatial scales^8,15^, requiring spatio-temporal models capable of simulating the spatial and temporal fire patterns. Several studies at EUMed have attempted to assess the ongoing evolution of the roles that biophysical conditions and human factors play in fire occurrence (presence/absence) and frequency (number of fires per unit area and time)^16–20^ from a stationary temporal point of view. In addition, other studies at EUMed have applied different models for various time periods and later comparing them over the longer term^21–25^. However, models that do not assume stationarity in the controlling variables (e.g., panel or longitudinal models) can be most useful^26–30^. On the other hand, to account for spatial heterogeneity (spatial non-stationarity), local Geographically Weighted Regression (GWR) has been commonly applied^18,20,23^. Nevertheless, to explicitly model spatial heterogeneity, hierarchical, multilevel modeling or the General Linear Mixed Models (GLMM) are also appropriate. These models have been successfully applied for predicting fire occurrence^31–33^. Finally, for dealing with high number of zero observations, zero-inflated models have been used for predicting fire spatial patterns^4,34–38^.

In this work, we modeled the variation of wildfires in space and over time by applying longitudinal Negative Binomial (NB) and Zero-Inflated Negative Binomial (ZINB) mixed models over a thirty year period, using wildfires of three size categories (≥1–10 ha, >10–100 ha, >100 ha) that occurred in a large rural area in west-central Spain. The objective was to determine the relationship between occurrence and frequency of wildfires as a function of various factors, including topography, LULC types and their changes, socio-economic variables, forest interfaces, linear features and climate in a dynamical way. We expected that wildfires would have increased in the area and during the study period due to changes in some of the main fire drivers: i) an increase in landscape hazardousness as result of LULC changes (i.e., land abandonment, afforestation, etc.), ii) socio-economic restructuring in the area due to rural exodus (i.e., population decline, shifts in economic activities), and iii) changes in climatic conditions (i.e., warming).

## Methodology

### The Study area

**Figure 1:**
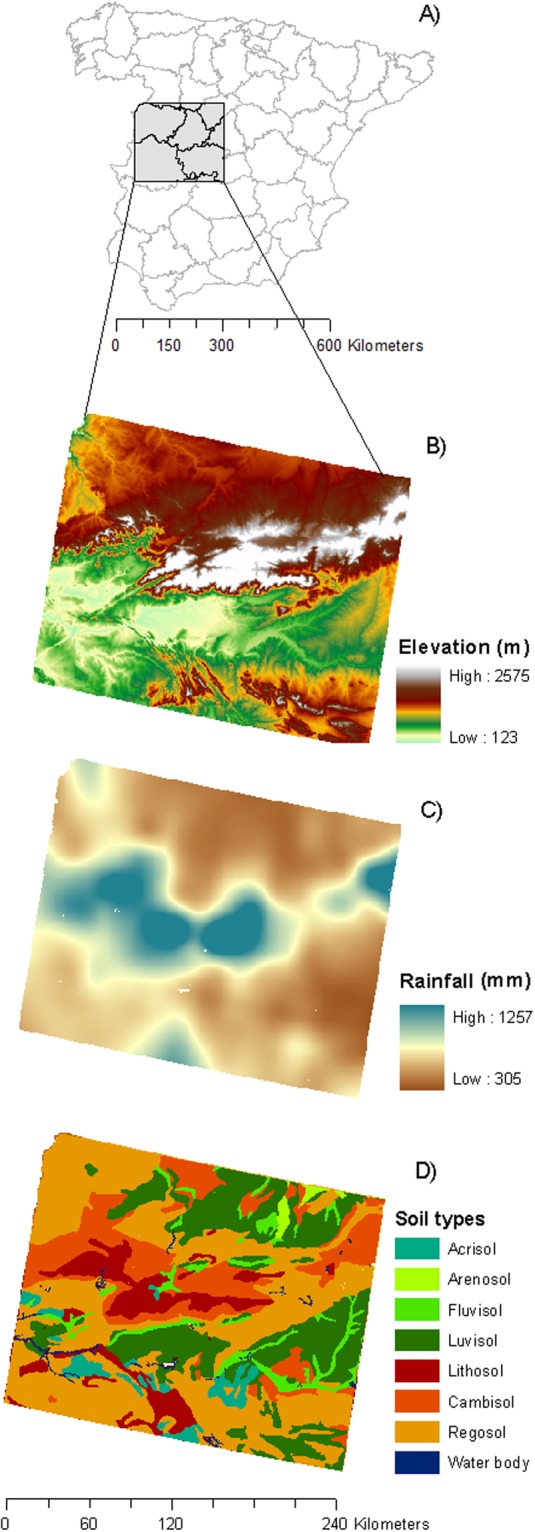
Location of the study area in west-central Spain (**A**); map of elevation range (**B**); map of mean annual rainfall (**C**); map of soil types (**D**).

The study area covers 56,000 km^2^ in west-central Spain (UTM coordinates 4369–4551 and 201–394 30 N) (Fig. 1A). The area is characterized by the mountainous landscapes of Sierra de Gredos, and the gentler mountains at the SE, both flanked by relatively flat areas (Fig. 1B). The climate is Mediterranean, with colder and wetter conditions up in the mountainous areas, and warmer and drier at the low-lands (Fig. 1C). Soils in the mountain areas are shallow, with high stoniness and coarse texture (cambisol, regosol and lithosol), whereas, in the low-lands, they are deep and fine textured (luvisol and fluvisol) (Fig. 1D).

### Fire data and explanatory variables

We used the yearly number of fires recorded at 10 × 10 km grid cells (n = 443) of the Spanish Fire Statistics database (EGIF, Ministry for the Ecological Transition), from 1979 to 2008 (30 years, n = 13,290 cells [443 × 30]). We selected fires ≥1 ha, and defined three fire size categories: small (≥1 to 10 ha); medium (>10–100 ha) and large (>100 ha). Most fires were lighted by people. The variables used to explain number of fires per cell per year included: topography (elevation, slope, radiation), landscape features (i.e., LULC [land-use-land cover] types and their changes [i.e., afforestation, agricultural abandonment, etc.], forest interfaces [wildland-urban interface, WUI; wildland-agrarian interface, WAI; wildland-grassland interface, WGI], linear infrastructures [roads, railways]), agrarian characteristics and socio-economy (farm size and density, agrarian holders [age, percent time dedicated], population density, employment), and climate (maximum temperature, the drought index SPEI, i.e., Standardized Evaporation-Precipitation Index, and Fire Weather Index of the Canadian system, FWI) (see Supplementary Material online, SM Table 1). These variables proved to be important in explaining wildfires in other areas^12,13,18,19^. An exploratory analysis based on Principal Components (PCA) was carried out to assess collinearity among explanatory variables (i.e., co-variates). Moreover, trends over time of the number of fires per cell for each fire size category and of each explanatory variable were assessed by means of the Mann-Kendall test (*Kendall* package^39^ in R software^40^). Additionally, we assessed the significance of the proportion of cells that changed over the years against the null hypothesis of an even proportion of positive/negative or no change, by a G test of goodness of fit^41^. The datasets generated during the current study are available in the figshare repository 10.6084/m9.figshare.7159727.

### Statistical approach

#### Longitudinal NB and ZINB mixed models

In gridded fire data, like the ones used here, fire counts are often characterized by a high number of zero observations, as no fires may occur in some years in a high number of spatial units. The excess of zeros yields a variance greater than the mean (over-dispersion)^42^. To handle over-dispersion in count data, a Negative Binomial (NB) distribution may be appropriate. Moreover, when in addition to over-dispersion an “excess of zeros” may be present, Zero-Inflated Negative Binomial (ZINB) models are theoretically most suitable^43^. ZINB models are composed of two parts: 1) a logistic regression for the “excess zeros” over and above what would be predicted by the count distribution, and 2) a regression (either Poisson or Negative Binomial) for the counts part^42,44^. Likewise, the number of fires per year at a given cell (i.e., fire frequency) is a type of longitudinal data with repeated measures, in which the observations are taken over time on the same subject (i.e., 10 × 10 km grid cells), thus observation are not independent. In these cases, longitudinal NB or ZINB mixed models handle dependency by incorporating an appropriate random component in the model structure^45–47^. To evaluate suitability of one modelling approach over the other the Vuong statistic^48^ was used. However, we did not find strong evidence to conclude that one model fitted the data better than the other^49^, and thus we decided to test both NB and ZINB models.

#### Modelling strategy

**Figure 2:**
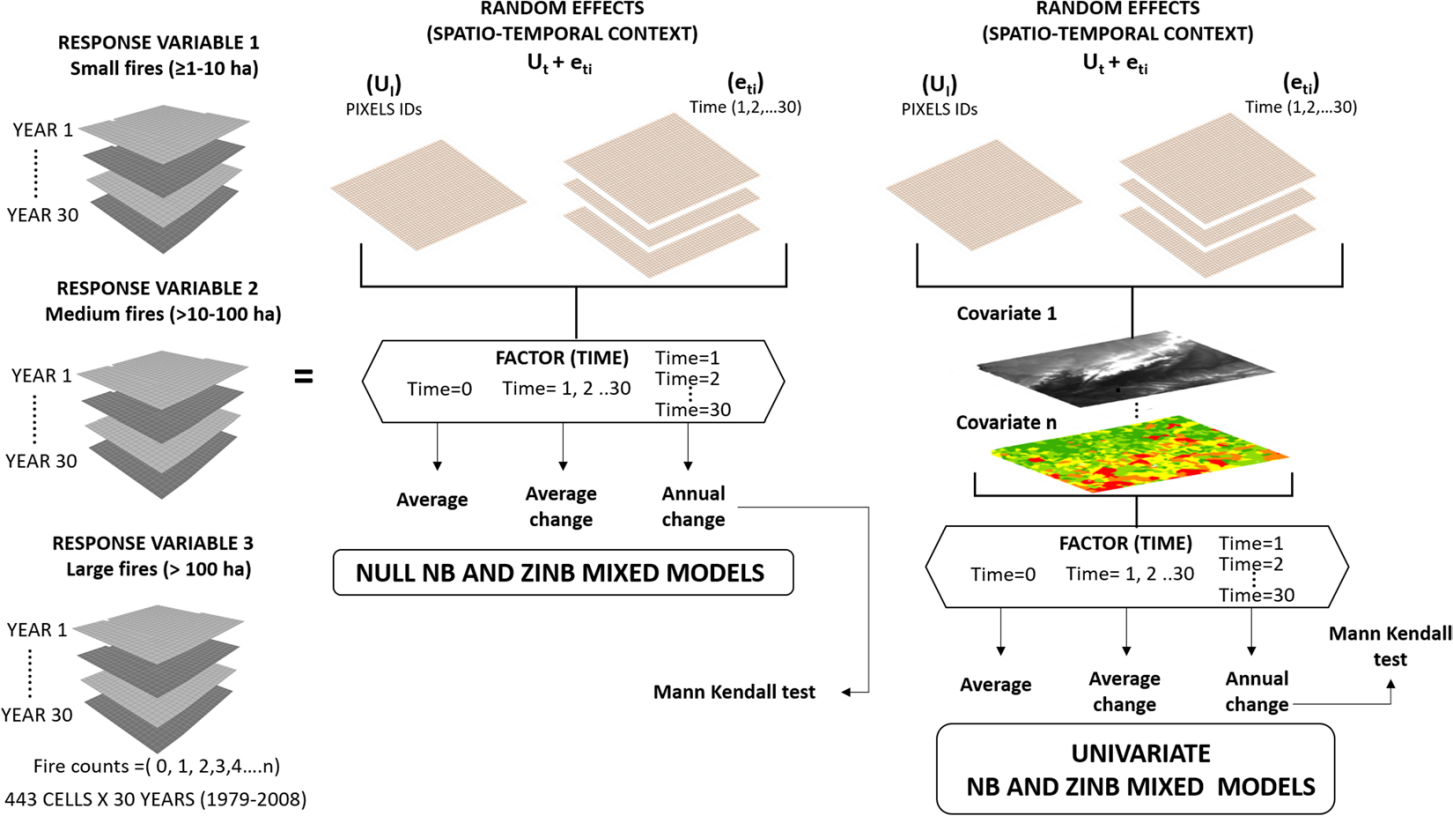
Flow of the strategy to model wildfires per 10 × 10 km cell per year of increasing size (≥1–10 ha,> 10–100 ha, >100 ha), by using longitudinal Negative Binomial (NB) and Zero-Inflated Negative Binomial (ZINB) mixed models in west-central Spain from 1979 to 2008.

We applied longitudinal NB and ZINB mixed models to three fire size categories (small [≥1–10 ha], medium [>10–100 ha], and large [>100 ha]) in order to explain the number of fires per cell per year as a function of several co-variates fitting univariate models (Fig. 2). To account for the spatial and temporal dependencies of our data, two random components were included: (i) time (T, years) as level 1 (repeated measures), which represents the “within-subject variance”, and (ii) an identifier for each grid cell as level 2 (subjects$$)$$, which represents “between-subject variance”^45^. In addition, to capture changes over time in the response variable and co-variates, time (T) was additionally included as interacting factor. This was done in three different ways, either it was not considered (T = 0), or considered as a continuous (T = 1, 2, …, 30) or categorical (T = 1; T = 2; …, T = 30) factor. This permitted obtaining three types of models, respectively: (i) ”Average” models, which provided the average response and the average effects of co-variates over the whole period; (ii) “Average change” models, which provided the initial response and the initial effect of each co-variate, as well as the average rate of change (positive or negative) over the years; and (iii) “Annual change” models, similar to the previous models except that they provided coefficients of change between the initial year and any other given year separately. “Annual change” models permitted a finer analysis of the trends, which were later explored by means of a Mann-Kendall test (Fig. 2). Null models (without covariates, thus being pure random effects models) provided the baseline to assess the performance of univariate models for each co-variate.

Model outputs were: intercept, slope of the time factor, and logit coefficient of the co-variates. The logit-coefficients, which were standardized by z-score and were conditional on the random effects, represent the following: in the “Average” models, the average effect of covariate (X) over the response variable (Y) when X changes between subjects and across time by one standard deviation (SD) unit^47^. In “Average change” and “Annual change” models, the regression coefficients of X were also conditional terms and must be interpreted along with the interaction term (X*Z [time interaction term])^46^. Finally, the logit coefficients were exponentiated to obtain the Odds Ratio (OR) and the Rate Ratio (RR). The OR represents the increasing or decreasing odds of the excess of zeros in the outcome (Y) for the zeros part in ZINB mixed models, and the RR represents the percentage increase or decrease in the outcome (Y) in NB mixed models and in the count part of ZINB mixed models. Fire occurrence (presence/absence) was assessed by the intercepts and coefficients of the zeros part in ZINB mixed models and fire frequency (number of fires) by the intercepts and coefficients of NB mixed models and those of the count part in ZINB mixed models.

#### Model evaluation and goodness of fit

Longitudinal NB and ZINB mixed models were calculated based on a Bayesian approach, using strong normal priors (V = diag (2) and nu = 4) after a sensitivity analysis. The posterior distribution of the model parameters were obtained using the Markov Chain Monte Carlo (MCMC) method, whose accuracy was assessed by density plots, autocorrelation analysis and the Geweke diagnostic^50^. Furthermore, we assessed the accuracy of the longitudinal NB and ZINB mixed models by linear regressions between the observed versus predicted values, using annual data (n = 13,290), proportion of zeros and counts correctly estimated, and by spatial overlays between predicted and observed values using aggregated (whole period) data. All models were calculated using the MCMCglmm package^50^ in R software^40^.

Given the large number of models, we focused on those univariate NB and ZINB mixed models that had a reduction [<−10] of DIC (Deviance Information Criterion), showed significant regression coefficients of the co-variates (p < 0.05), and explained >20% of random variance relative to that of null models (pseudo-R^2^). For each model, we checked that Markov chains did not show autocorrelation or any patterning in parameter estimations.

## Results

### Changes in wildfires and drivers

**Figure 3:**
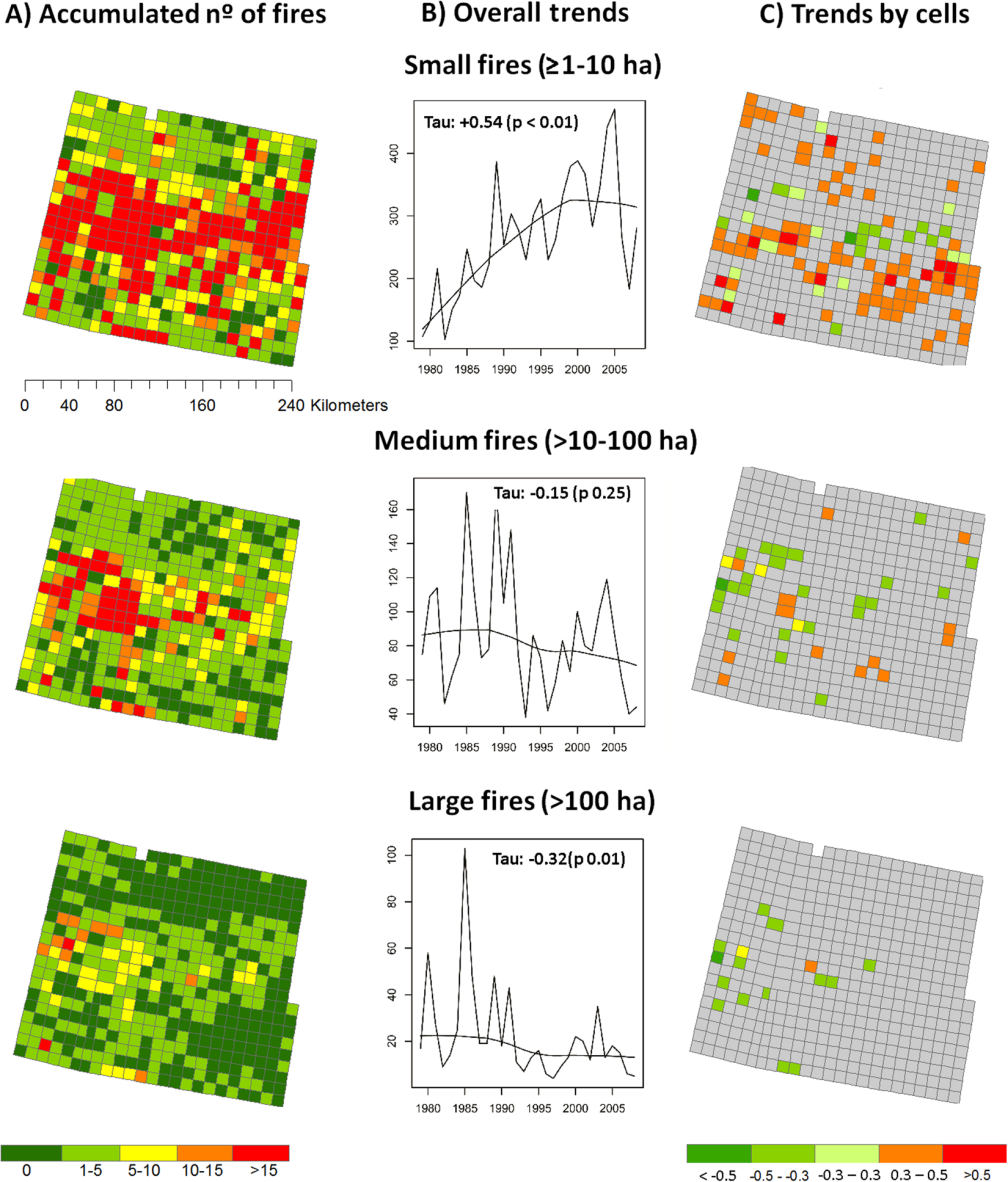
Accumulated number of fires aggregated by time (1979–2008) (**A**); temporal trends of the number of fires aggregated spatially (all cells) applying a Mann- Kendall test (**B**); temporal trends of the number of fires by cell (**C**). Upper panels correspond to small fires (≥1–10 ha), middle panels to medium fires (>10–100 ha), and bottom panels to large fires (> 100 ha). In grey, the cells with no significant temporal trend.

During the 30-yr period, there were 23,435 fires, which burned a total of 456,253 ha. Number of fires per cell over the study period varied between 0 and 326. Annually, several cells contained a high proportion of zeros, thus the distribution was heavily left skewed (74%, 88% and 96% of total annual counts were zeros for fires ≥1–10 ha, 10–100 ha and >100 ha, respectively). The three fire size categories accounted for 34%, 11%, 3% of all fires, and 7%, 18%, and 74% of the burned area, respectively (Fig. 3A). In the whole area, temporal trends of small fires showed a significant increase (Mann-Kendall Tau: +0.54, p < 0.001), no significant trend was obtained for medium fires, and a significant decreasing trend for large fires (Tau: −0.32, p < 0.01) (Fig. 3B). Temporal trends at cell level indicated that 26%, 4% and <1% of cells showed a significant positive trend, whereas 3.8%, 8% and 7% of cells showed a significant negative trend for each fire size, respectively (Fig. 3C).

PCA of the explanatory variables showed that these could be merged in 3 main axes (SM Fig. 1). Axis 1 (38% of total variance) was positively related to areas with high solar radiation and high density of livestock, and negatively related to high elevation and slope ranges, with hazardous vegetation (shrublands and dense forests), which experienced hazardous stability and hazardous LULC changes (i.e., densification and afforestation). Axis 2 (24%) was positively related to areas with herbaceous crops, crop-trees and WAIs, and negatively related to pastures, agroforestry, large farms, agriculture abandonment and WGIs. Axis 3 (19%) was mainly related to artificial uses, development changes (i.e., artificialization), population density, unemployment and WUIs.

Moreover, we found that landscapes in the study area were rather dynamic (SM Fig. 2 and SM Table 2). In general, landscapes became more hazardous over time due to the increase of hazardous stability (i.e., hazardous LULC types that remained so over time) thus accumulating fuel, the spread of open and broadleaved forests as well as pastures, which resulted in important spatial changes in forest interfaces. Other important changes were the appearance of development in former more natural areas, hence increasing WUIs; the spread of non-hazardous (i.e., herbaceous crops and agroforestry) over areas in which they were not so abundant, thus raising the WAIs; and the loss of abundance of some hazardous LULC types (i.e., coniferous forests, shrublands) in some cells due to wildfires^10^, which was compensated by increases in other cells not traditionally occupied by such LULC types at the beginning of the study. Regarding socioeconomic factors, we found: population increases in peri-urban areas around the main towns and cities; a reduction of employees in the primary sector, with an increase employment in the building sector; a significant reduction of farm density, together with a decrease of small farms and an increase of larger ones; a significant increase of livestock density and a reduction of agricultural machinery. Finally, in relation to climate, there was not any clear trend for any of the variables analyzed (SM Fig. 2).

### Wildfires modelling

**Figure 4:**
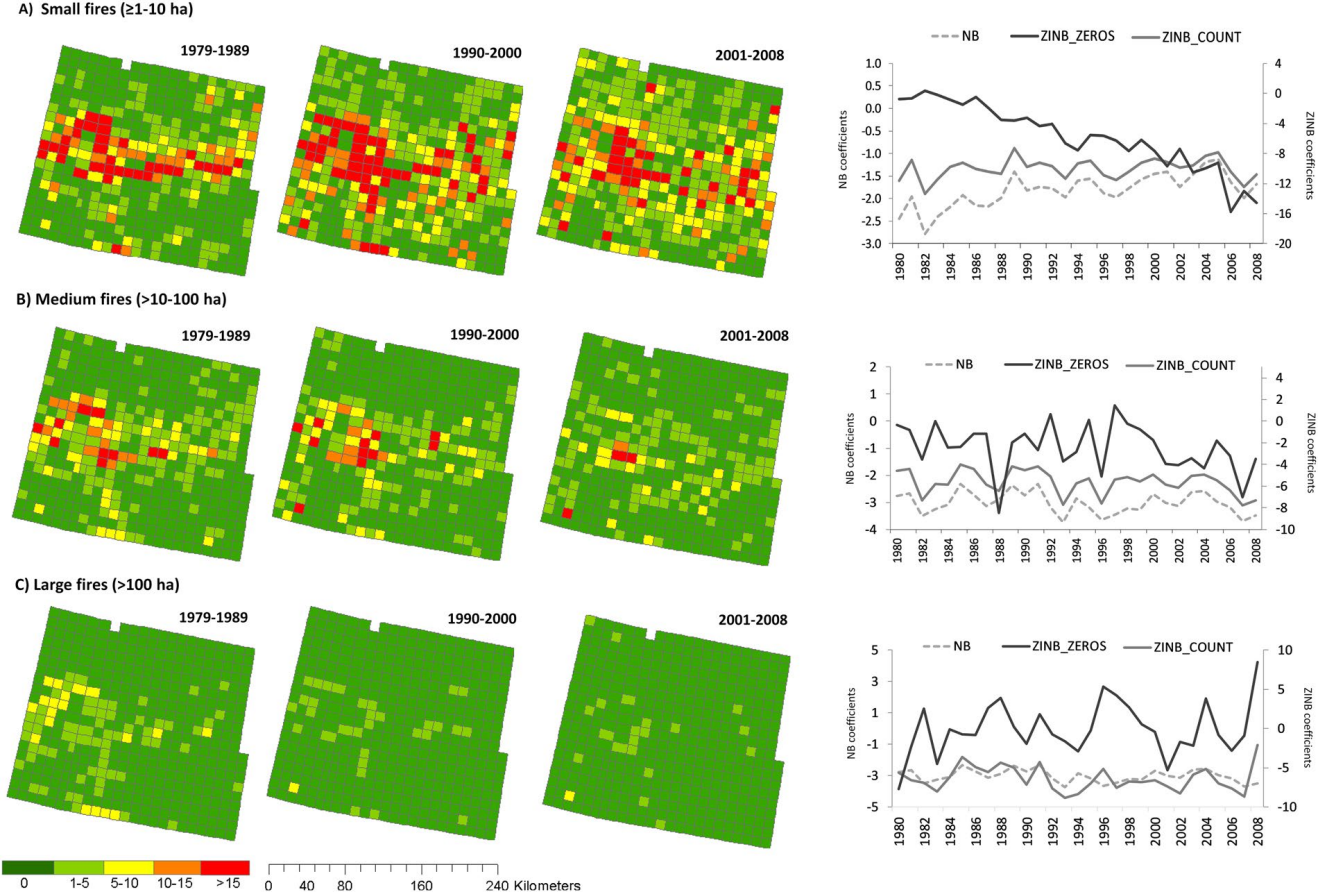
Spatial pattern of the number of fires per decades (1979–1989, 1990–2000 and 2001–2008) and temporal dynamic of logit intercepts [Ln (Intercept * Time categorical terms)] for the null “Annual change” longitudinal Negative Binomial (NB) and Zero-Inflated Negative Binomial (ZINB) mixed models. Upper panels correspond to small fires (≥1–10 ha) (**A**), middle panels to medium fires (>10–100 ha) (**B**), and bottom panels to large fires (>100 ha) (**C**).

The number of fires per cell per year was small at the beginning (1979) for all fire size categories, particularly for large fires, as is reflected in the low rate ratio of the intercepts of “Average change” null NB and ZINB mixed models. According to NB mixed models, small fires tended to increase over time, medium fires did not show any significant trend, and large fires decreased, as shown by the effect of time (Table 1). Similarly, time effects in the “Average change” null ZINB mixed models indicated that the probability of small and medium fire occurrence (presence/absence) increased over time (lower excess of zeros), whereas the frequency (number of fires per cell per year) of medium and large fires (counts part of the model) slightly decreased (Table 1, Fig. 4).

**Table 1:** Results of longitudinal null Negative Binomial (NB) and Zero-Inflated Negative Binomial (ZINB) mixed models for explaining number of fires of different size in west-central Spain from 1979–2008.

	Negative Binomial mixed models			Zero-Inflated Negative Binomial mixed models					
	Average	Average change	Annual change	Average		Average change		Annual change	
	Logit [RR]	Logit [RR]	Logit [RR]	zeros (Logit [OR])	count (Logit [RR])	zeros (Logit [OR])	count (Logit [RR])	zeros (Logit [OR])	count (Logit [RR])
***Intercept***									
≥1–10 ha	−1.83** [0.16]	−2.38** [0.09]	−2.75* [0.06]	−4.46** [0.01]	−1.27** [0.28]	1.91** [6.75]	−1.33** [0.26]	2.00 ns [7.41]	−1.53 ns [0.22]
10–100 ha	−3.01** [0.05]	−2.82** [0.06]	−3.11* [0.04]	−1.71 ns [0.18]	−2.28** [0.1]	0.17 ns [1.18]	−1.77** [0.17]	−1.13 ns [0.32]	−2.11 ns [0.12]
>100 ha	−4.62** [0.01]	−4.01** [0.02]	−4.68** [0.01]	0.82 ns [2.27]	−2.99** [0.05]	1.60* [4.96]	−2.16** [0.12]	2.48 ns [11.93]	−3.68 ns [0.03]
***Time effect***									
≥1–10 ha		0.04** [1.04]				−0.40** [0.67]	<0.01 ns [1.00]		
10–100 ha		−0.01 ns [0.99]				−0.15** [0.86]	−0.03* [0.97]		
>100 ha		−0.04** [0.96]				0.03 ns [1.04]	−0.05** [0.95]		
***Random effects***									
Spatial (≥1–10 ha)	2.10	2.10	2.11	30.25	1.61	24.69	1.67	29.89	1.73
Spatial (10–100 ha)	1.82	1.82	1.85	8.51	1.34	13.51	1.27	13.28	1.29
Spatial (> 100 ha)	2.01	2.07	2.05	8.72	0.85	12.40	0.68	15.65	1.26
Temporal (≥1–10 ha)	0.29	0.21	1.91	13.53	0.19	0.59	0.19	1.99	1.97
Temporal (10–100 ha)	0.28	0.28	2.46	1.37	0.29	1.51	0.26	1.79	2.18
Temporal (> 100 ha)	0.64	0.54	1.76	1.02	0.52	0.83	0.48	1.79	2.18
Residual variance (≥1–10 ha)	0.63	0.63	0.63		0.45		0.44		0.45
Residual variance (10–100 ha)	0.83	0.83	0.83		0.59		0.58		0.58
Residual variance (> 100 ha)	0.91	0.90	0.92		0.64		0.64		0.65
DIC (≥1–10 ha)	19573	19570	19568	19224		19248		19187	
DIC (10–100 ha)	10374	10375	10368	10436		10386		10388	
DIC (> 100 ha)	4132	4134	4132	4258		4248		4154	

Logit coefficients and their transformation in Rate Ratio (RR) (for NB models and count part of ZINB mixed models) and Odds Ratio (OR) (for the zero part in ZINB mixed models) (in brackets) are shown; the random effects (spatial [between subjects] and temporal [within subjects]) and residual variances, as well as the Deviance Information Criterion (DIC) are given.

**Figure 5:**
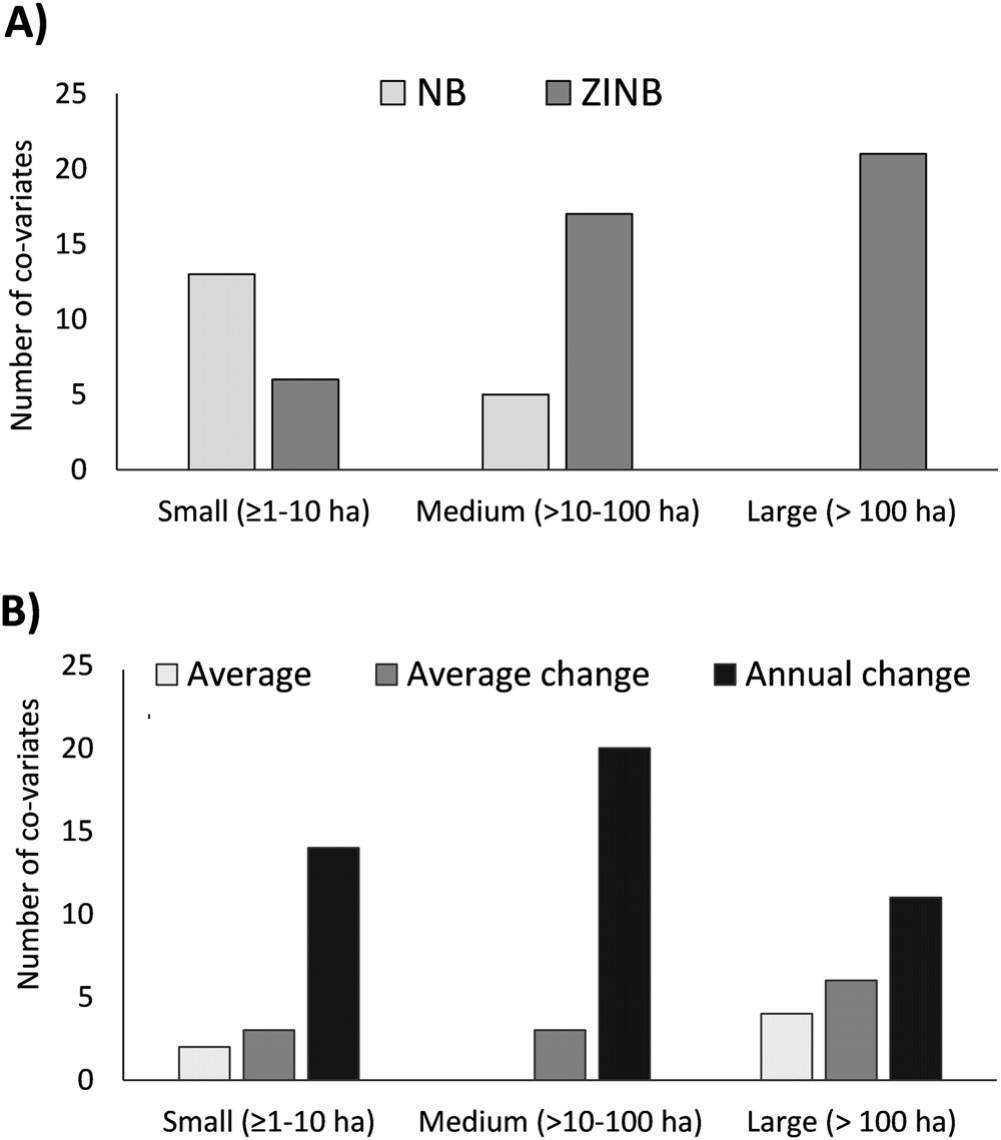
Number of co-variates with the highest DIC (Deviance Information Criterion) reduction in: longitudinal Negative Binomial (NB) and Zero-Inflated Negative Binomial (ZINB) mixed models (**A**) and in models in which time was not considered (“Average”) or considered as a continuous (“Average change”) or categorical (“Annual change”) factor (independently of whether they were NB or ZINB mixed models) (**B**).

**Figure 6:**
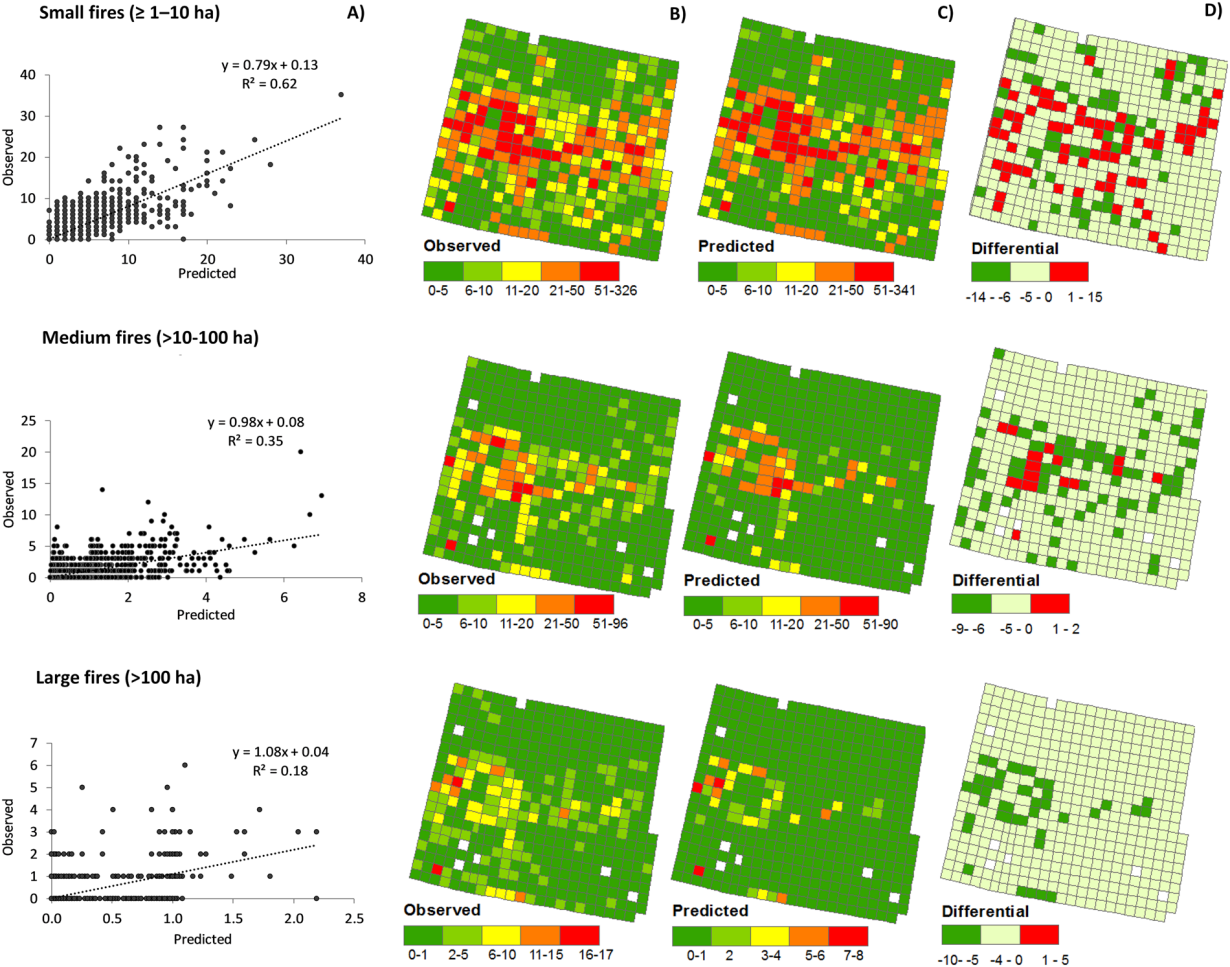
Linear regression between observed versus predicted number of fires averaged for all significant co-variates and univariate longitudinal NB and ZINB mixed models (**A**). Observed and predicted number of fires aggregated over time (1979–2008) for the same models (**B**,**C**). Spatial accuracy of predicted number of fires (difference between observed and predicted fires) (**D**). Small fires (≥1–10 ha) in upper panels, medium fires (>10–100 ha) in middle panels, and large fires (> 100 ha) in bottom panels.

Based on reduction of DIC, the best univariate models were the NB mixed models for small fires, and the ZINB mixed models for medium and, particularly, for large fires (Fig. 5A, Table 2). In relation to the way “time” was included in the models, “Annual change” models were always better than the other two models for all fire sizes, although for large fires, “Average” and “Average change” models were also appropriate (Fig. 5B). Linear regression between observed versus predicted values indicated that, on average, for all co-variates and significant models, small fires were better explained (R^2^ = 62%), followed by medium (R^2^ = 35%), and, to a lesser extent, by large ones (R^2^ = 18%) (Fig. 6A). Moreover, from ZINB mixed models we ascertained that the number of zeros was better predicted for both medium and large fires than for small ones (91% 103% and 69%, respectively), whereas the number of fires (counts) was better predicted for small fires than for medium and large fires (89%, 57%, 19%, respectively). Spatially, there was an error of ±15 fires per cell for small fires, −9/+2 for medium fires and −10/+5 for large fires (Fig. 6B–D).

**Table 2:** Best univariate NB and ZINB mixed models for modelling wildfires of different size (small [≥1 - 10 ha]; medium [>10–100 ha] and large [>100 ha]).

FACTORS	Small fires (≥1–10 ha)								Medium fires (10–100 ha)								Large fires (>100 ha)							
	NB models			ZINB models					NB models			ZINB models					NB models			ZINB models				
	Cf.	Tr.	DIC diff.	Cf. (zeros)	Tr.	Cf. (count)	Tr.	DIC diff.	Cf.	Tr.	DIC diff.	Cf. (zeros)	Tr.	Cf. (count)	Tr.	DIC diff.	Cf.	Tr.	DIC diff.	Cf. (zeros)	Tr.	Cf. (count)	Tr.	DIC diff.
**Topography**																								
Elevation range (m)	+	−	−156	−	+	+	−	−108	+	−	−64	ns	+	+	ns	−34	+	ns	−47	—	ns	ns	ns	−80
Slope range (degrees)	+	−	−201	−	+	+	−	−116	+	−	−76	−	+	+	ns	−57	+	ns	−42	−	ns	+	ns	−101
Solar radiation (hours)	−	+	−179	+	−	−	+	−176	−	+	−80	+	−	ns	ns	−131	−	ns	−35	+	ns	−	ns	−124
***Land-use/Land cover types***																								
Conifer forests (%)	+	−	−175	−	+	+	−	−34	+	−	−39	ns	ns	ns	−	−123	ns	ns	−23	ns	ns	+	ns	−74
Broadleaf forests (%)	.	.		.	.	.	.		+	ns	−13	−	ns	+	ns	−150	.	.		.	.	.	.	
Mixed forests (%)	+	−	−53	−	ns	+	−	−14	.	.		.	.	.	.		.	.		.	.	.	.	
Open forests (%)	ns	−	−51	−	+	−	ns	−91	.	.		.	.	.	.		.	.		.	.	.	.	
Shrublands (%)	+	−	−89	−	+	ns	ns	−32	+	ns	−42	−	ns	ns	ns	−115	.	.		−	ns	+	ns	−29
Grasslands (%)	.	.		.	.	.	.		.	.		.	.	.	.		.	.		−	ns	−	+	−32
Agroforestry (%)	.	.		.	.	.	.		−	ns	−14	−	ns	—	ns	−125	ns	ns	−24	ns	ns	−	ns	−28
Crop-trees (%)	.	.		.	.	.	.		.	.		.	.	.	.		.	.		ns	ns	+	ns	−23
Herbaceous crops (%)	−	+	−157	+	−	−	+	−54	−	+	−55	+	ns	−	ns	−123	−	ns	−33	+	ns	ns	ns	−68
***Land-use/Land cover changes***																								
Hazardous stability (%)	+	−	−218	−	+	+	ns	−84	+	−	−64	−	+	+	ns	−136	+	ns	−21	−	ns	ns	ns	−41
Agriculture abandonment (%)	.	.		.	.	.	.		.	.		.	.	.	.		.	.		−	ns	+	ns	−49
Hazardous densification (%)	.	.		.	.	.	.		+	−	−25	−	+	ns	ns	−125	.	.		.	.	.	.	
Hazardous afforestation (%)	+	−	−100	−	ns	+	−	−62	+	−	−24	−	ns	+	−	−71	ns	ns	−11	−	ns	ns	ns	−44
Agriculture conversion (%)	−	+	−156	+	−	−	+	−93	−	+	−26	ns	ns	−	ns	−59	.	.		.	.	.	.	
Artificialization (%)	.	.		ns	−	+	+	−64	ns	ns	−11	−	ns	ns	ns	−68	.	.		−	ns	ns	ns	−46
***Agrarian characteristics***																								
Farm density (n°/km^2^)	.	.		.	.	.	.		+	−	−17	−	+	+	ns	−79	.	.		.	.	.	.	
Farms <5 ha (%)	+	−	−60	−	ns	+	ns	−54	+	ns	−35	ns	ns	+	ns	−95	+	ns	−14	−	ns	+	ns	−15
Farms> 50 ha (%)	−	+	−62	+	ns	−	+	−12	−	+	−44	ns	ns	−	+	−126	ns	ns		ns	ns	−	ns	−49
Farms in property (%)	+	−	−38	−	ns	+	−	−48	+	−	−48	.	.	.	.		.	.		.	.	.	.	
Farms in leasing (%)	−	+	−26	+	ns	−	+	−41	−	+	−34	.	.	.	.		.	.		ns	ns	−	ns	−47
Agric. machines (n°/Km^2^)	−	+	−66	ns	ns	−	+	−16	.	.		.	.	.	.		.	.		.	.	.	.	
***Socio-economy***																								
Full-time farmers (%)	−	+	−26	ns	ns	−	+	−44	.	.		.	.	.	.		.	.		.	.	.	.	
Old farmers (%)	.	.		.	.	.	.		+	ns	−15	.	.	.	.		.	.		+	ns	ns	ns	−25
Population density	.	.		.	.	.	.		−	+	−20	+	ns	ns	+	−103	ns	ns	−30	+	ns	−	ns	−22
***Forest interfaces***																								
Wildland−agrarian (WAI) (%)	.	.		.	.	.	.		.	.		.	.	.	.		.	.		+	ns	+	ns	−44
Wildland−grasslands (WGI) (%)	.	.		.	.	.	.		.	.		.	.	.	.		.	.		−	ns	−	ns	−53
Wildland-Urban (WUI) (%)	.	.		.	.	.	.		.	.		ns	ns	+	ns	−69	ns	ns	−18	−	ns	ns	ns	−23
***Climate***																								
Max. summer temp.	.	.		.	.	.	.		−	ns	−15	+	ns	−	ns	−36	.	.		.	.	.	.	
SPEI (April-May-June)	.	.		ns	ns	−	ns	−41	−	ns	−10	.	.	.	.		.	.		.	.	.	.	

Models included were those whose regression coefficients were significant (p < 0.05), Deviance Information Criterion DIC < −10 and explained random variance >20%; ns: regression coefficient non-significant. Generally, best models corresponded to “Annual change”. For NB models: Cf., refers to the sign of the regression coefficient; Tr.: time interaction with significant trend ascertained by Mann-Kendall test; DIC diff., difference relative to the null models. For ZINB models, coefficients are provided for the zeros (Cf. [zeros]) and count part (Cf. [count]). Variables that did not reach the criteria set for model performance in a particular model/fire size combination are represented with a dot. The variables that did not reach these criteria for any combination are listed next: artificial surfaces, livestock, employees in primary and building, unemployment, roads and railways and Fire Weather Index (FWI).

### The role of fire drivers on wildfires

Some of the co-variates were able to largely reduce DIC and explain a large part of the random variance (pseudo-R^2^) (SM Tables 3–8). In general, topography, LULC types, LULC changes and agrarian characteristics were the group of variables that were more related to fires of all sizes, although with differences depending on fire size (Table 2, SM Tables 3–8). We found that topographic features were strongly linked to fire occurrence and frequency for all fire sizes, particularly for large fires. High fire occurrence (low excess of zeros) and frequency were linked to high elevation and slope ranges, whereas in low elevation areas with high radiation occurred the opposite. Regarding LULC types, fires of all sizes were positively related to conifer forests, which mainly explained fire occurrence of small fires, and fire frequency of medium and large ones. Similarly, shrublands were positively related to the occurrence of all fire sizes, particularly for small and medium fires. On the contrary, herbaceous crops were negatively linked to fires of all sizes, mainly large fires. Regarding to LULC changes, hazardous stability and afforestation explained positively fires of all sizes, whereas agriculture conversion showed the opposite. Concerning socio-economic variables, agrarian characteristics showed that large farms, usually under leasing, were negatively related to fires of all sizes. Population and employment variables were weakly related to fires. Finally, forest interfaces, linear structures and climate variables were marginal in explaining fires (Table 2, SM Tables 3–8).

### Changes through time in the role of fire drivers

The changing role through time of fire drivers on wildfires was ascertained using Mann Kendall test on the logit coefficients of “Annual change” NB and ZINB mixed models (Table 2). Regarding topography (SM Fig. 7), areas with high elevation and slope ranges showed lesser probability of having fires (greater number of zeros) over time. In contrast, areas with high radiation (i.e., flatter areas) showed the opposite trend. Regarding LULC types (SM Fig. 8), coniferous forests tended to loose statistical weight (coefficients in zeros part tended to zero) for explaining fire occurrence for all fire sizes, and showed negative trends for fire frequency regardless of size. Additionally, shrublands tended to have less fire occurrence of small fires over time, not showing significant trend for medium and large ones. Conversely, herbaceous crops showed a clear trend to increase the probability of occurrence of small fires, and reduced their statistical weight for explaining fire frequency of fires of all sizes (coefficients tended to zero in the count part). More to, areas with hazardous stability and afforestation showed less probability of fire occurrence and less fire frequency, respectively, over time for small and medium fires. On the contrary, agriculture conversion showed a positive trend for the frequency of small and medium fires. Similarly, large farms reversed its sign, increasing fire frequency with time (SM Fig. 9).

## Discussion

This study documents that the studied landscape of west-central Spain was rather dynamic. Wildfires were also very dynamic, as they changed in time and space, changes which were size dependent: small fires increased and spread over the landscape, while medium and large were stable or decreased. Models used to explain wildfires were also size dependent, with small fires being better explained by NB and larger fires by ZINB. Time was an important factor, and including it in the models as a categorical variable improved model performance. Not all sets of variables were able to equally explain wildfires, with topography, LULC types and their changes, and agrarian characteristics producing the best models. Within these sets, several variables had similar model performance, suggesting high collinearity between them. Additionally, the role of variables in explaining fires changed along the time, so that variables that were important at the beginning of the study period were not so at the end. Following, we discuss these findings in detail.

We found that the landscape in west-central Spain was rather dynamic. The main changes were characterized by the maintenance of hazardous LULC types, an increment in open forest and grasslands, as well as spatial changes in forest interfaces. The observed LULC changes were compatible with an increase in landscape fire hazard. For example, the increment in open forests and grasslands likely contributed to enhance fuel continuity. Furthermore, the increase in forest interfaces, notably WAIs, elevated the accessibility of ignitions to forest and other flammable areas. These processes were similar to those described in other Mediterranean countries^7,10,51^. Such changes have often been related to increases in wildfires^52,53^.

Wildfires were also dynamic, but this was fire size dependent. Whereas small fires increased over time and became widespread much over the whole landscape, medium fires remained stable and large fires decreased. Medium and large fires exhibited a spatial concentration, since they tended to disappear from areas in which they were present at the beginning of the study period, without spreading to new areas. The fact that the number small fires tended to increase suggests that ignitions were facilitated by a landscape with greater number of interfaces. Despite of that, and the more hazardous landscape that has evolved with time, large fires tended to decrease, which supports that firefighting services are rather efficient at deterring fires^9^. Notwithstanding, when conditions are severe, very large, even megafires, are erupting in the region^54^, supporting that there is limit to what the firefighting services can accomplish when conditions pass certain threshold, during which extant landscape can’t but facilitate fire spread. Our findings are compatible with wildfire trends across southern European Mediterranean-type countries. Indeed, wildfires have been decreasing in these countries^3,6,55^. This tendency, however, varies among countries and within countries. For example, while the reduction of fires and area burned in Spain, France and Italy are clear, that is not so much the case with Portugal^54,55^. Moreover, within Spain, while in a majority of provinces wildfires have been decreasing, in other provinces, including the ones comprehended in our study area, were increasing, mostly due to the contribution of small fires^5,56^.

ZINB mixed models explained better the occurrence (presence/absence) of medium and large fires and the frequency of small fires. Moreover, ZINB mixed models substantially reduced the variance in the random components, after incorporating the zero-inflated part^44^, particularly for small and large fires. ZINB models have been previously used to model wildfires^33,35–37^; although other works that used ZINB and NB models found no evidence to favor the first over NB models^34^. Additionally, including time as categorical interacting factor improved model performance for all fire sizes. Notwithstanding, average models were also appropriate for large fires. Applying zero-inflated models to longitudinal data, including random effects, should be further considered to study wildfires in dynamic landscapes like the Mediterranean ones.

Of the seven sets of variables used for modelling wildfires, four of them were the ones producing best model results, which is supported by the literature: topography^57–60^, land use-land cover^20,25,61^ and its changes^7,18,51,62^, and agrarian characteristics^18,33,53,63^. Other fire drivers as socio-economy, forest interfaces and linear features showed lower explanatory power for all fire sizes, which confirms other works^64^, even if these findings are not general^12,19,22,24,65^. Additionally, we could not ascertain an effect or trend of climate variables, despite changes underwent by some of them in southern Europe in recent decades^66^. On the other hand, there were large differences in model performance of these sets of variables, depending on fire size and modelling approach. While topographic variables had the best model performance for large fires (largest DIC reduction), other sets of variables produced similar results for small and medium fires (e.g., LULC changes types and their changes). Moreover, within a given set of variables, not any one variable produced the best results (highest DIC reduction) across fire sizes and either for NB or ZINB models. This permits arguing that this area is very complex from a perspective of wildfires^61,67^. Fires are ignited by people, with varying sensibilities and needs, and with changing capacities to fight fire over time.

In addition, the role of the various variables changed over time. Initially, wildfires were concentrated in mountainous terrain, where farming was limited and the vegetation was dominated by conifers and other hazardous types, which underwent important changes (e.g., afforestation) over time. Wildfires were abundant in landscapes with smaller farms, and rare in areas with larger ones. By contrary, fires were limited where agricultural activities dominated, proper of the low elevation and flatter areas. With time, some variables decreased their statistical weight such as in conifer forest, or elevation, the latter being so mainly for medium and large fires. Other variables reversed their sign, as shrublands, hazardous stability and large farms. The net result was that wildfires moved to areas at lower elevation, with herbaceous crops and large size farms. Our findings support studies that found that topographic variables tended to have lower contribution to explain wildfires in the last decades^16^, as well as those studies that found that wildfires have decreased on forests^22,65^ as well as on shrublands^25,68^, while increased on less flammable areas^22^.

To conclude, the initial link of wildfires to areas of certain hazardous characteristics, in which they abounded at the beginning of the study period, was lost. This process has also been observed in other Mediterranean areas of Greece^52^ and Portugal^68^, in which recent fires have moved partially to non-fire-prone areas, indicating a departure from historical burning patterns. This again supports arguing that the complex nature of this human dominated landscapes, in interaction with the ignitions generated by people, complicates understanding of how fires will evolve over time and in space. In this regard, deterministic modelling approaches to infer future fires due to climate change or other global change drivers will be plagued with uncertainties. Consequently, if projections of future fires are difficult to make in general^69^, in Mediterranean landscapes, like the one studied, will be even more challenging.

## Electronic supplementary material

Supplementary Material
