# Supplementary material for: Wildfires and the role of their drivers are changing over time in a large rural area of west-central Spain
Source: Sci Rep. 2018 Dec 12;8:17797. doi: 10.1038/s41598-018-36134-4 (PMC6290888; doi:10.1038/s41598-018-36134-4)
Supplement: Supplementary file 1 — Supplementary Material [file 41598_2018_36134_MOESM1_ESM.docx]

**Wildfires and the role of their drivers are changing over time in a large rural area of Central Spain**

**Viedma, O.*, Urbieta, I.R. and Moreno, J.M.**

*Departamento de Ciencias Ambientales*

*Universidad de Castilla-La Mancha*

*Avda. Carlos III, 45071 Toledo, Spain*

# ***SUPPLEMENTARY MATERIAL***

# **Methodology**

# ***Relevance of explanatory variables***

The variables used to explain wildfires per cell per year for each fire size category during the 30-yr period were the following: topography, LULC types and their changes, socio-economy, forest interfaces, linear infrastructures and climate (SM Table 1). All of these had been used on similar type of works (Martínez et al. 2009; Vilar del Hoyo et al. 2011; Martínez-Fernández et al. 2013; Rodrigues et al. 2014a; 2014b, among others). Some of these works have detailed explanations about the role of these variables on wildfires, either directly (i.e., topography and climate are related to conditions for fire propagation; LULC types are related to fuels; WUIs are related to ignitions) or indirectly (i.e., infrastructure represents accessibility to the territory, and thus probability of ignitions since most ignitions are related to humans; unemployment can be related to abandonment or LULC changes, that is, ultimately to fuels).

# ***Description of the explanatory variables***

*Topography*: We used a 5 m Digital Terrain Model (DTM) resampled at 100m (IGN, Spanish National Geographic Institute). The following topographic variables were derived for each 10x10 km cell using ArcGis 9.3.1. ([www.esri.com](http://www.esri.com)): elevation (m), slope (º) and solar radiation duration [hours]).

*Landscape features*: The National Land-Use/Land-Cover Map of 1978 and the National Forest Map of 1986 (http://www.mapama.gob.es), as well as the 2000 and 2006 maps of CORINE Land Cover project (<http://www.eea.europa.eu/>) were combined to derive: i) main Land Use/Land Cover (LULC) types for 1980s, 1990s, 2000 and 2006, ii) LULC changes (between consecutive dates), iii) forest interfaces, and iv) linear features (roads and railways). For comparative analyses, all maps were equally referenced (WGS 84 UTM 30 N) at 100m of spatial resolution. A common LULC legend based on CORINE (Bossard et al. 2000) was applied to all maps: croplands (herbaceous and woody crops), agroforestry areas or “*dehesa*s” (i.e., open oak- woodlands), pastures (natural and artificial herbaceous vegetation), shrublands (evergreen bush and scrub), open forests (< 30% tree cover), dense forests (separating deciduous, conifer and mixed forests, with > 30% tree cover), developed or artificial uses (urban, industrial) and water bodies. The main LULC changes considered were those contributing to increase landscape fire hazard (i.e., agriculture abandonment, densification of shrublands and open forests, afforestation with conifers, and hazardous stability [unchanged LULC types of pastures, shrublands and open and dense conifer and mixed forests, that persisted through time, thus accumulating fuels]). Furthermore, LULC changes that contributed to reduce fire hazard were also considered: development (changes from any LULC type to artificial uses) and agriculture intensification (from any LULC type to agriculture) (for further details see Viedma et al. 2015). Interfaces were calculated from buffers of 400 m around wildland patches (open and dense forests and shrublands). Urban, croplands, pastures and agroforestry areas were intersected with the wildland buffers to create the wildland-urban (WUI), wildland-agrarian (WAI) and wildland-grasslands (WGI) interfaces, respectively. Finally, roads and railway density (km/km^2^) at each cell was obtained from buffers of 100 m around them.

*Socioeconomic variables*: Population density at municipality level was derived for 1981, 1991 and 2001 from the Spanish Annual Population Register (Institute of National Statistics, INE). Labour-forces, i.e., employees in different activity sectors (mainly primary and building sector), as well as unemployed population were obtained from the Population Censuses for the same years. Data about farm and farmers’ characteristics was available at municipality level from the Agrarian Censuses (INE) of 1982, 1989 and 1999. The main variables derived were: density of farms; proportion of farms by size classes (<5 ha [small farms] and > 50 ha [large farms]); proportion of farms by land tenure (property and leasing); proportion of agrarian holders older than 55 years; and livestock and machinery density.

*Climate and fire weather:* Each cell was characterized by: summer (June-September) maximum temperature (Tmax), derived from Spain02 Gridded Dataset (Herrera et al. 2016); the Standardized Precipitation-Evapotranspiration Index (SPEI: SPEIbase v2.2) (Beguería et al. 2014) accumulated from March to May, that is, the three months (SPEI03) antecedent to the fire season; and mean summer FWI (Fire Weather Index, van Wagner, 1987), derived from the WATCH-Forcing-Data-ERA-Interim (Weedon et al. 2014).

# ***Spatial Analysis***

The spatial analysis applied to derive these covariates to a common spatial unit of 10x10 km (the spatial unit of the wildfire data) consisted on: 1) For continuous variables as topography, we intersected both layers (the variable of interest and the grid of 10 x 10 km) and calculated the statistical value (mean and range) for each cell. 2) For variables which were percentages of occupation within each cell (i.e., LULC types, LULC changes, interfaces and linear infrastructures), we intersected those layers with the regular grid, and later, we calculated the area occupied by those features in each cell. 3) For statistical variables (i.e., socio-economy variables derived at municipality scale), the spatial analysis consisted on intersecting the polygons of municipalities with the grid-cells after which, we assigned to each cell the statistical value that corresponded to the overlapping municipality. When several municipalities overlapped a grid cell, a weighted average by the area occupied by each municipality in the grid was assigned to the cell. All time-variant variables were arithmetically interpolated between each census and between maps to derive annual data.

**SM Table 1.** Variables used for explaining wildfires in the various modeling approaches.


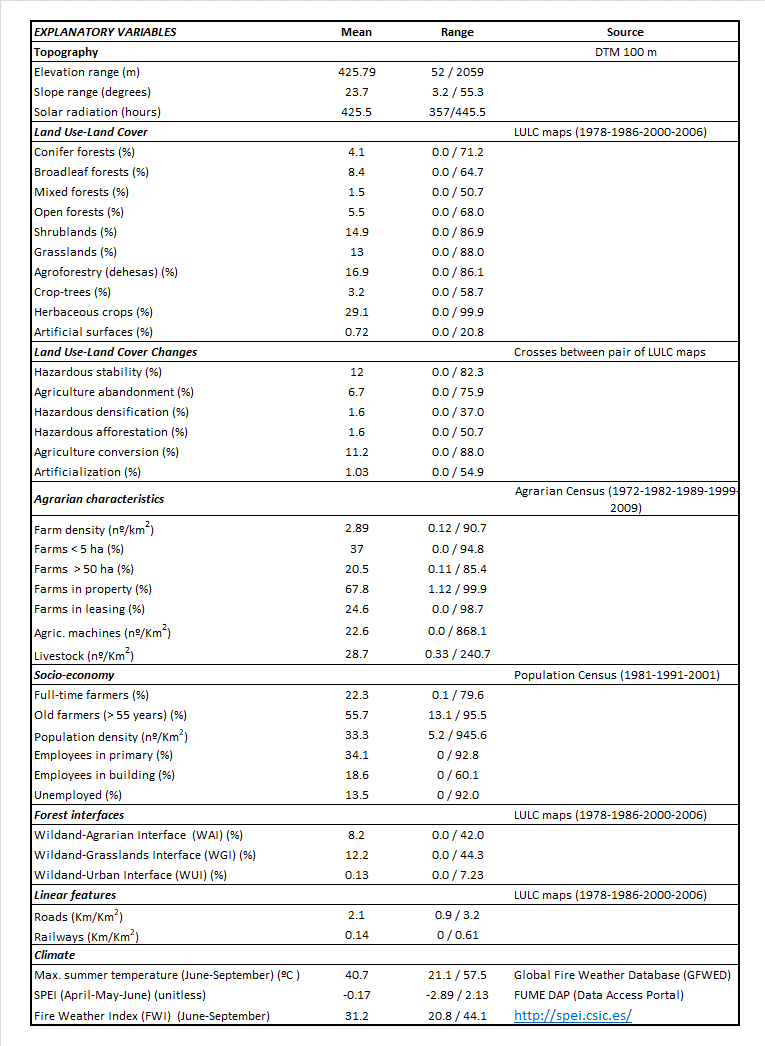


# ***Results***


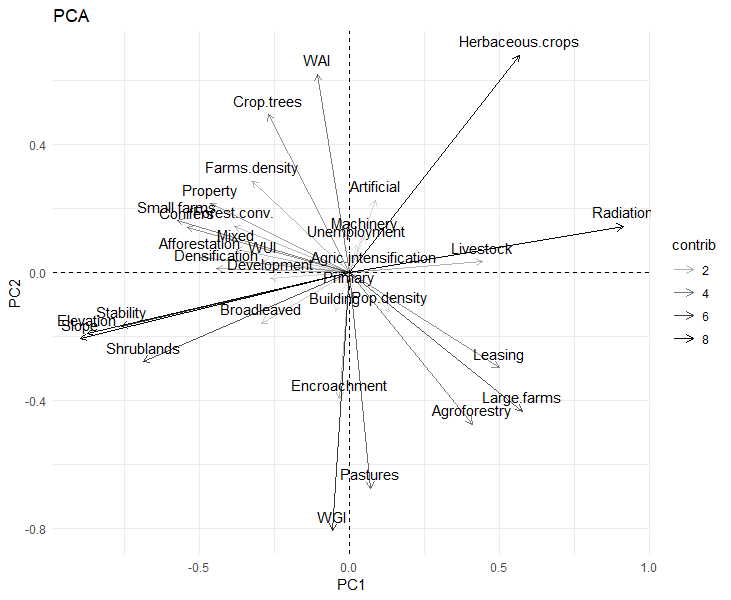


**SM Figure 1.** Principal Components Analysis (PCA) of fire drivers averaged over the study period (1979-2008). The three first axes explained 38%, 24% and 19% of total variance, respectively


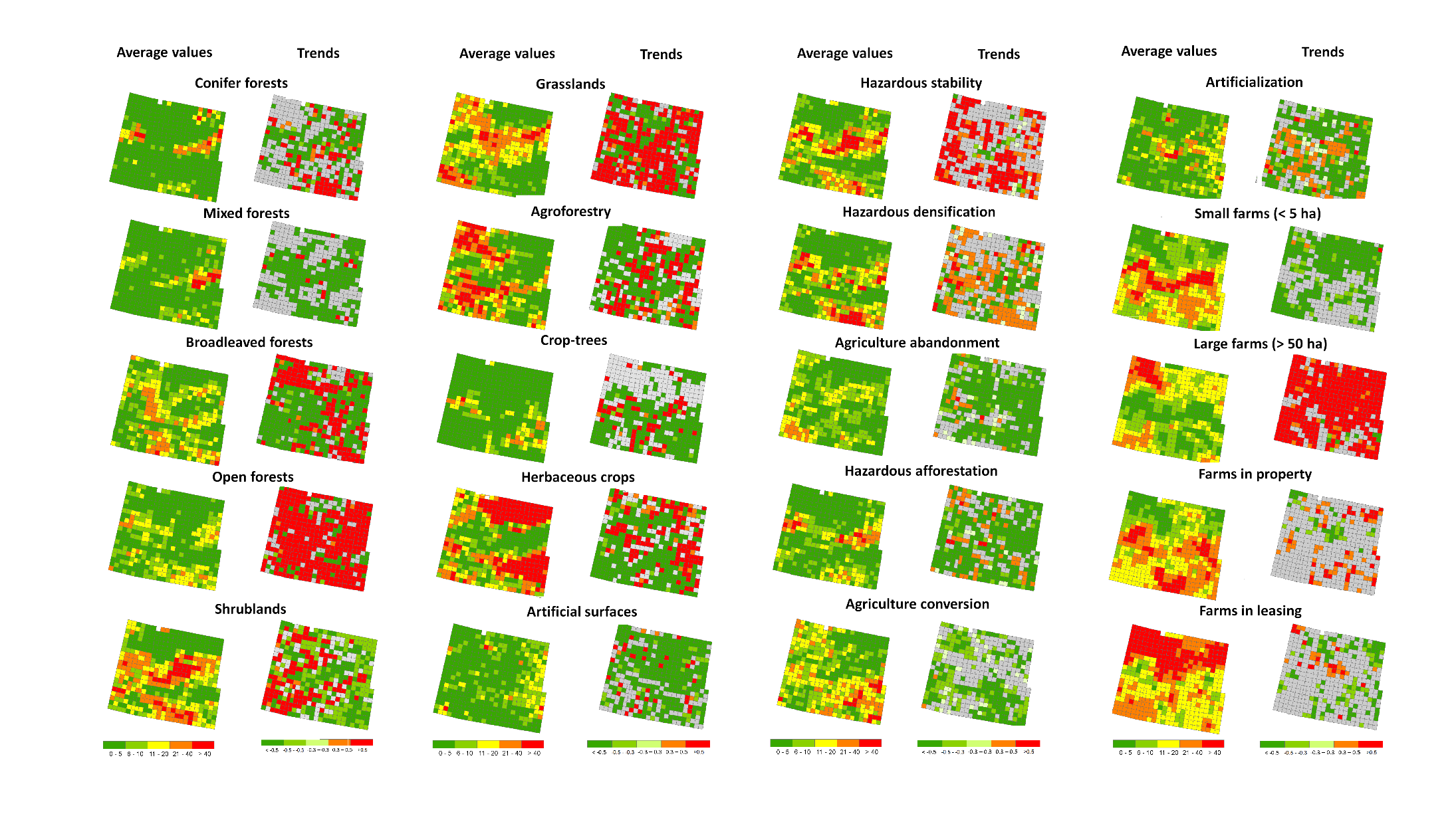


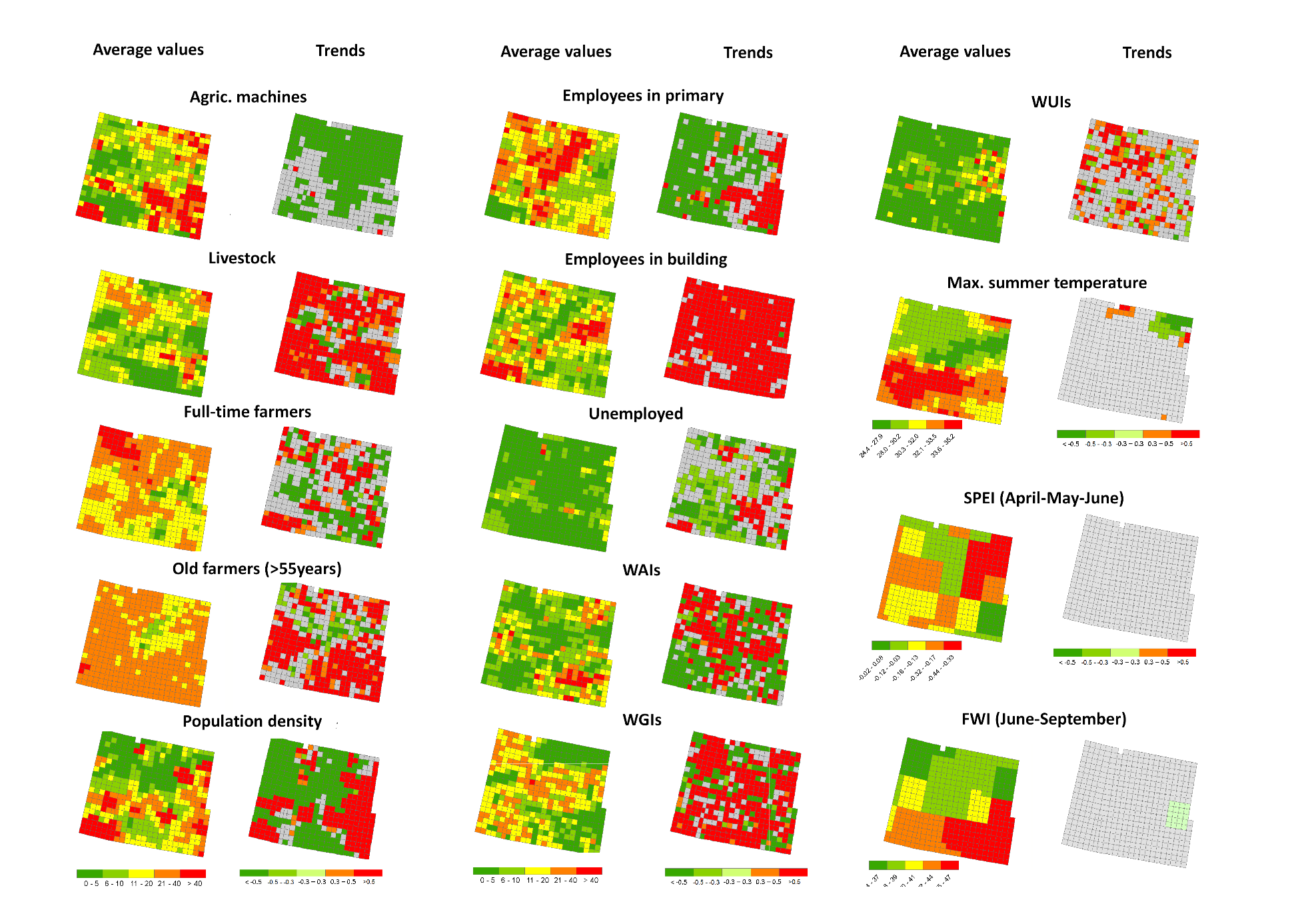


**SM Figure 2.** Average values (1979-2008) (see SM Table 1 for units) and trends (Tau, Mann Kendall test) (grey colors denote non-significant trends) for the main variables used in longitudinal NB and ZINB mixed models of wildfires in West-Central Spain.

**SM Table 2.** Percentage of cells (10 x 10 km) occupied by the main fire drivers that changed (positively or negatively) or not changed over time (by Mann Kendall test) and their significance, according to the G-test of goodness of fit, in West-Central Spain during the period 1979-2008.


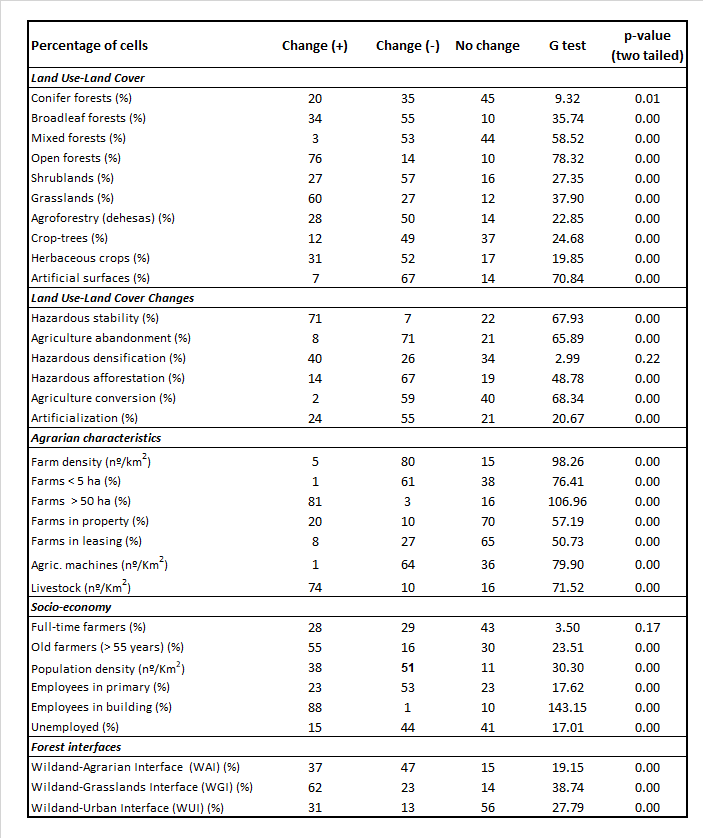


**SM Table 3**. Results of the longitudinal univariate Negative Binomial (NB) mixed models for explaining the number of small fires (≥1-10 ha) in West-Central Spain from 1979-2008. Logit coefficients for co-variates and time, pseudo-R^2^ and DIC difference relative to the null models are given. Significant coefficients (p < 0.05) are in bold.


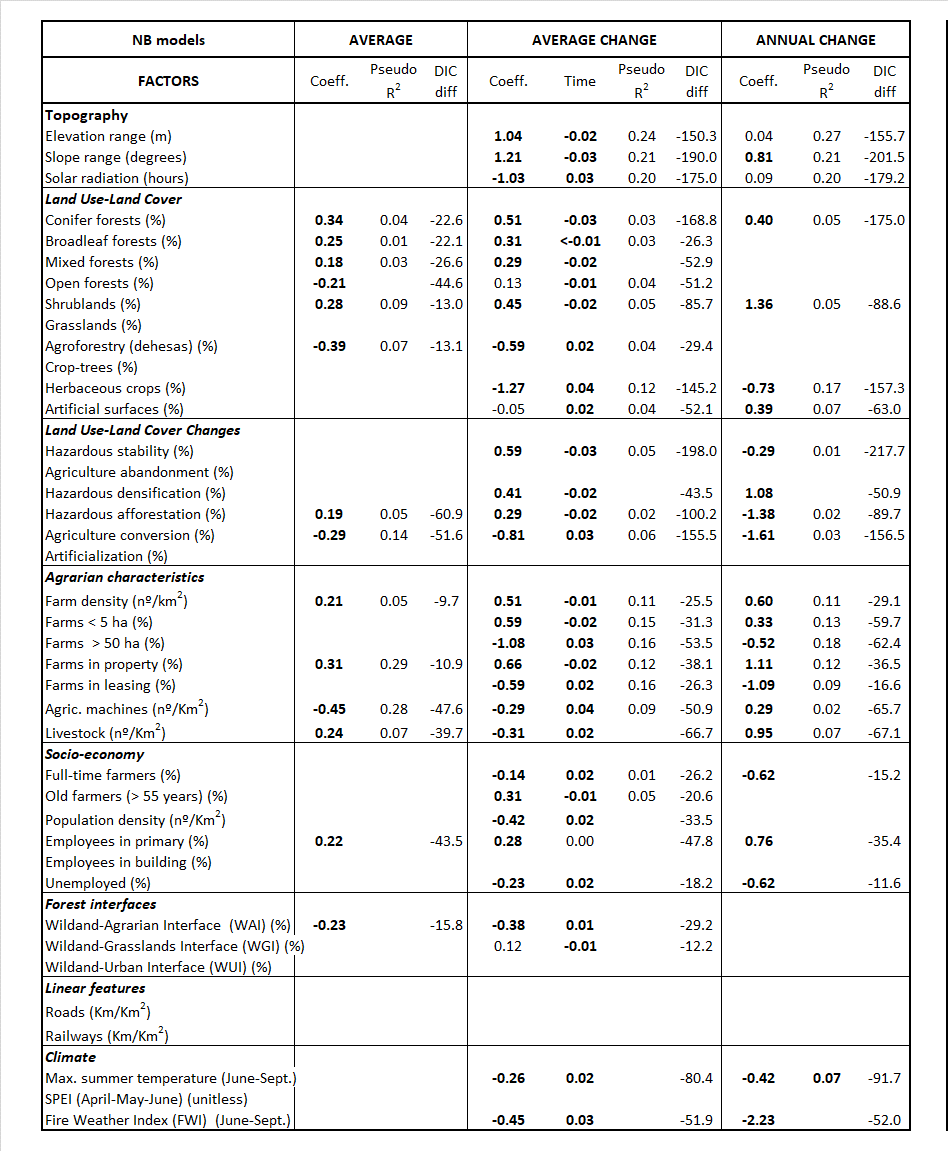


**SM Table 4.** Results of the longitudinal univariate ZINB mixed models for explaining the number small fires (≥1-10 ha) in West-Central Spain from 1979-2008. Logit coefficients for co-variates and time, pseudo-R^2^ and DIC difference relative to the null models are given. Significant coefficients (p < 0.05) are in bold.


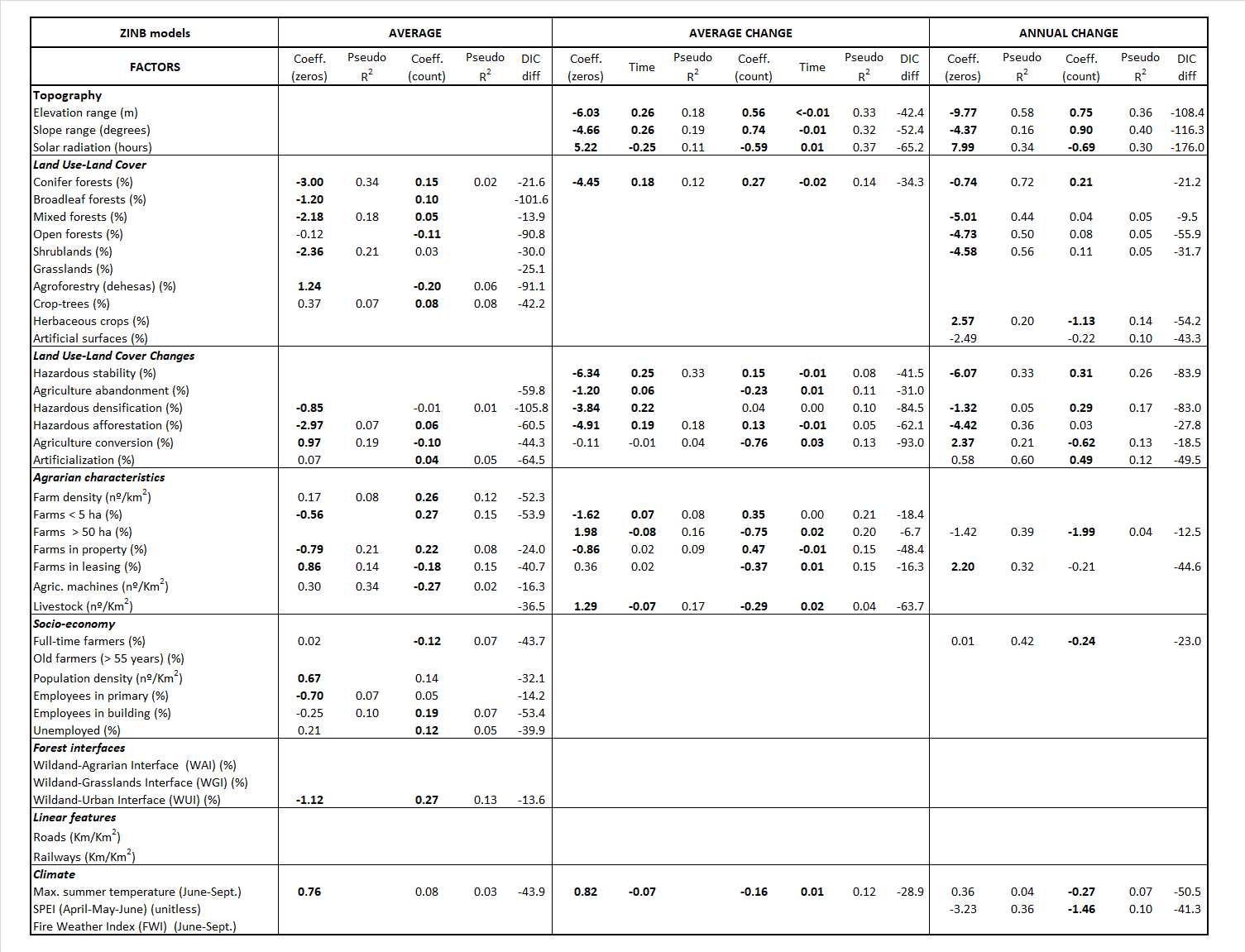


**SM Table 5.** Results of the longitudinal univariate Negative Binomial (NB) mixed models for explaining the number of medium fires (>10-100 ha) in West-Central Spain from 1979-2008. Logit coefficients for co-variates and time, pseudo R^2^ and DIC difference relative to the null models are given. Significant coefficients (p < 0.05) are in bold.


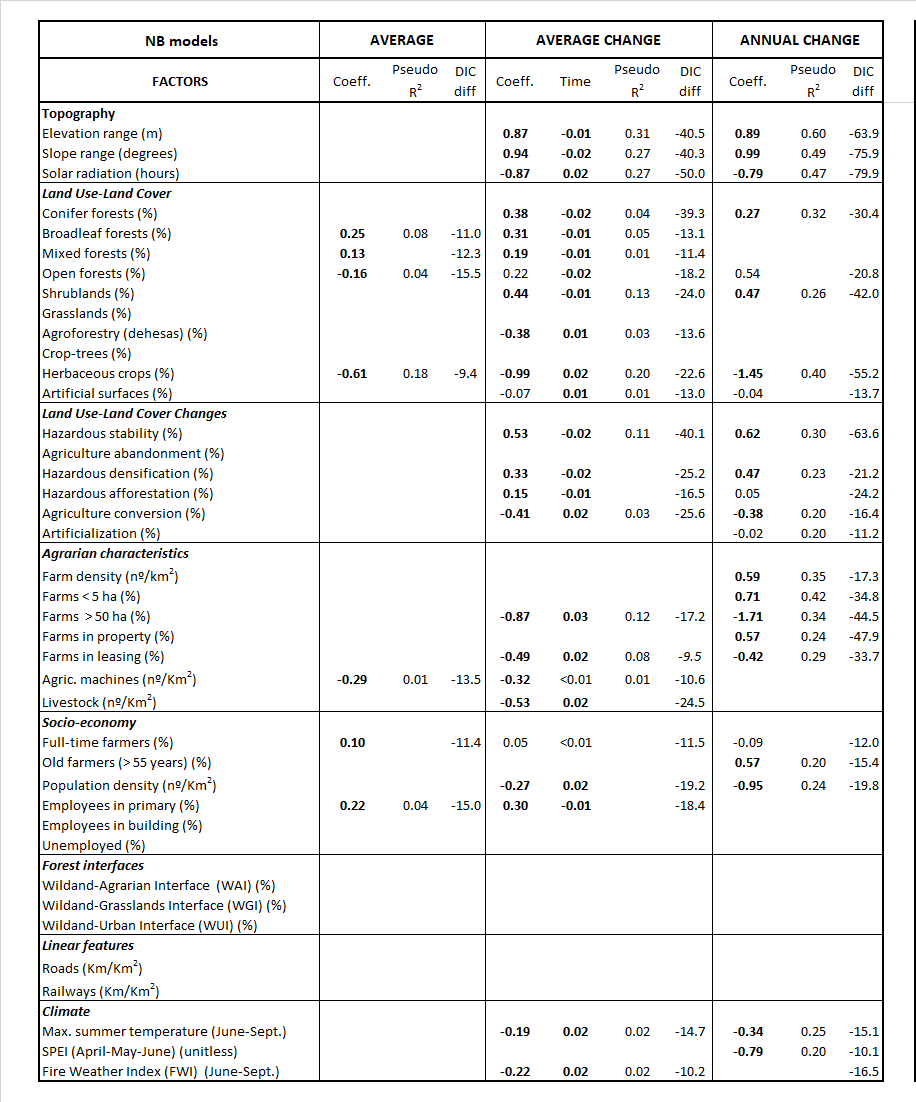


**SM Table 6.** Results of the longitudinal univariate ZINB mixed models for explaining number of medium fires (>10-100 ha) in West-Central Spain from 1979-2008. Logit coefficients for co-variates and time, pseudo R^2^ and DIC difference relative to the null models are given. Significant coefficients (p < 0.05) are in bold.


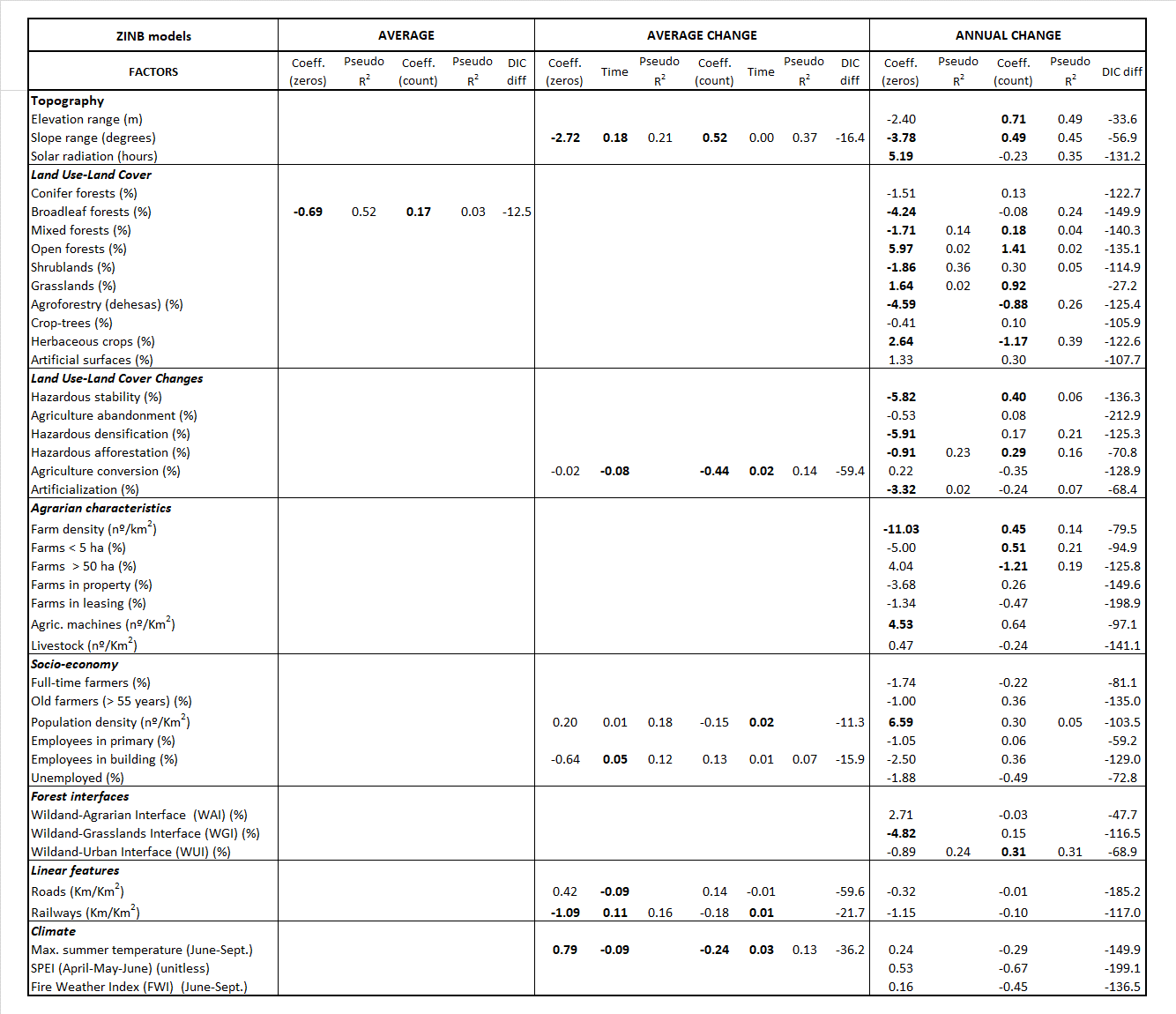


**SM Table 7.** Results of the longitudinal univariate Negative Binomial (NB) mixed models for explaining number of large fires (>100 ha) in West-Central Spain from 1979-2008. Logit coefficients for co-variates and time, pseudo R^2^ and DIC difference relative to the null models are given. Significant coefficients (p < 0.05) are in bold.


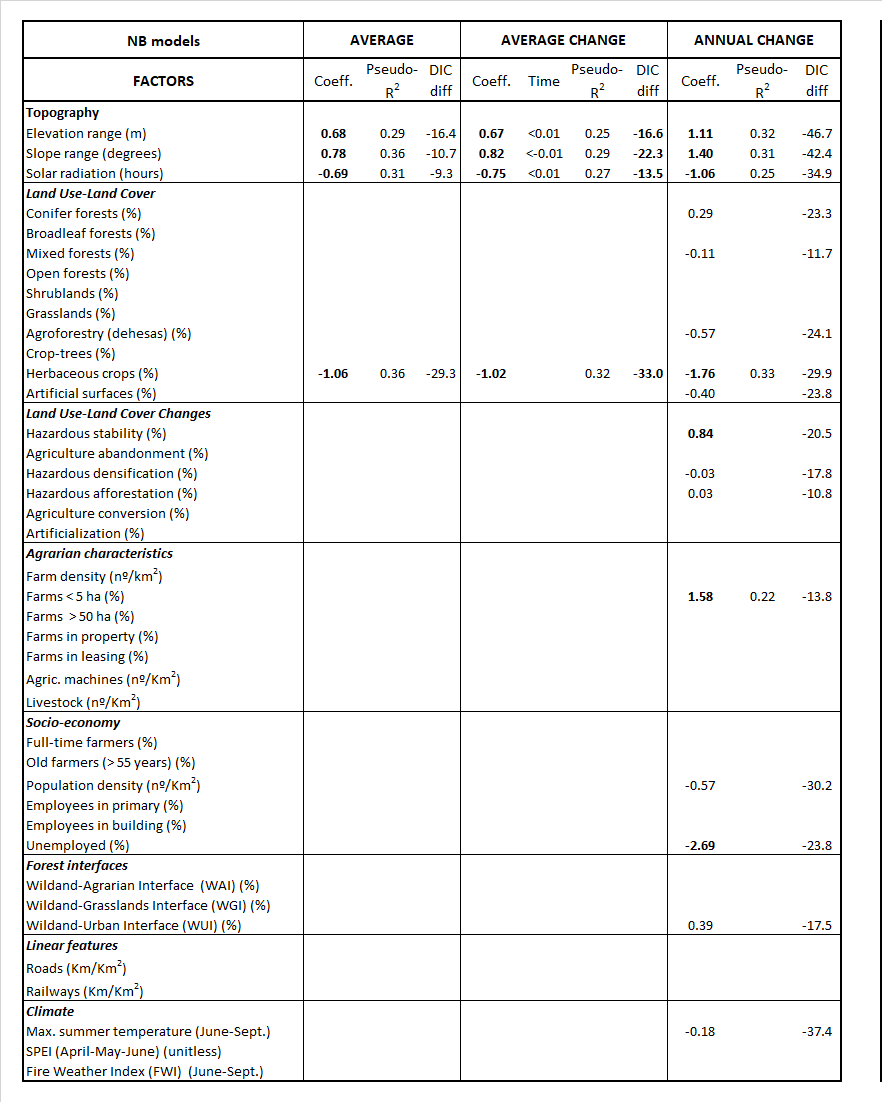


**SM Table 8**. Results of the longitudinal univariate ZINB mixed models for explaining number of large fires (>100 ha) in West-Central Spain from 1979-2008. Logit coefficients for co-variates and time, pseudo R^2^ and DIC difference relative to the null models are given. Significant coefficients (p < 0.05) are in bold.


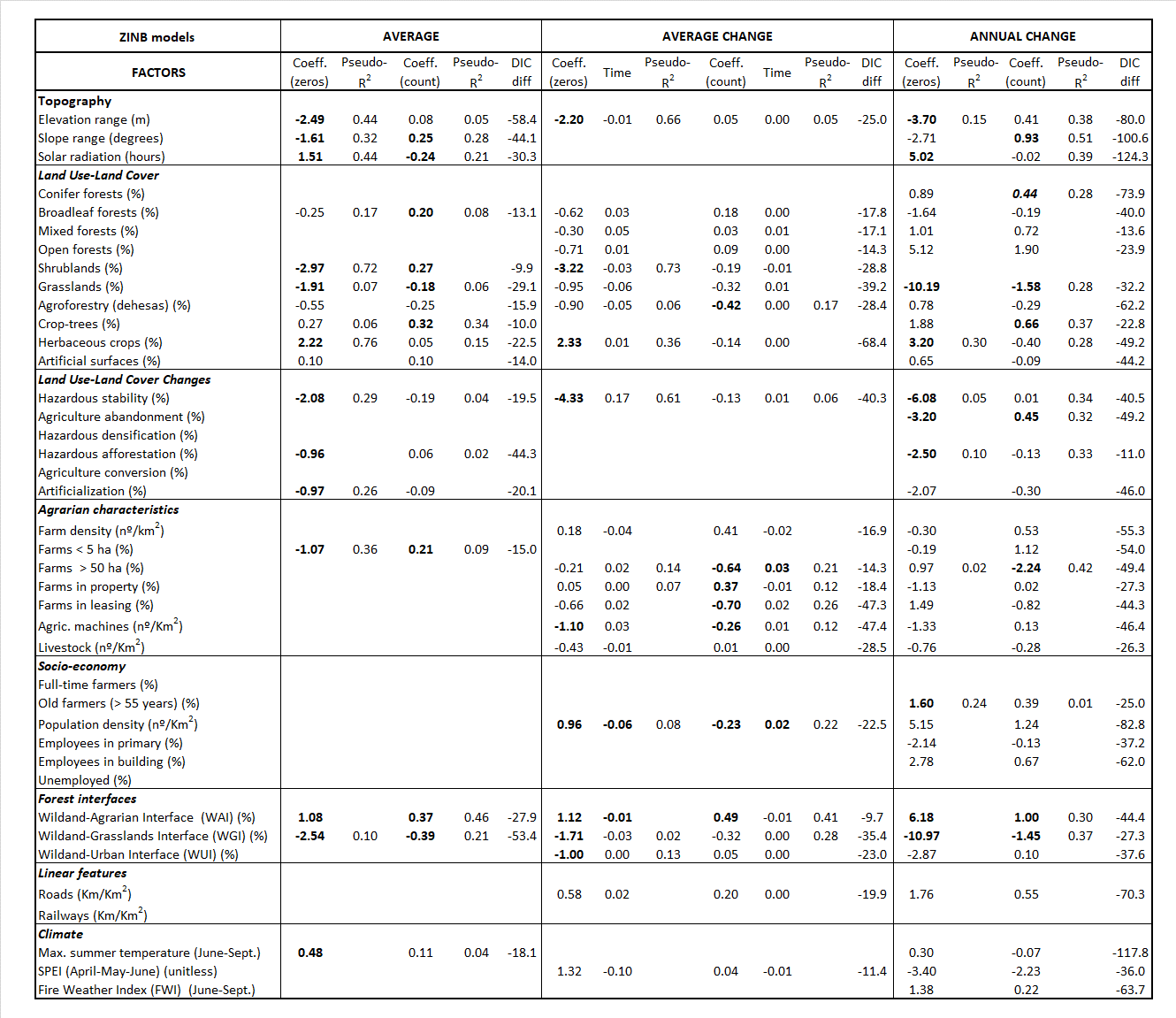


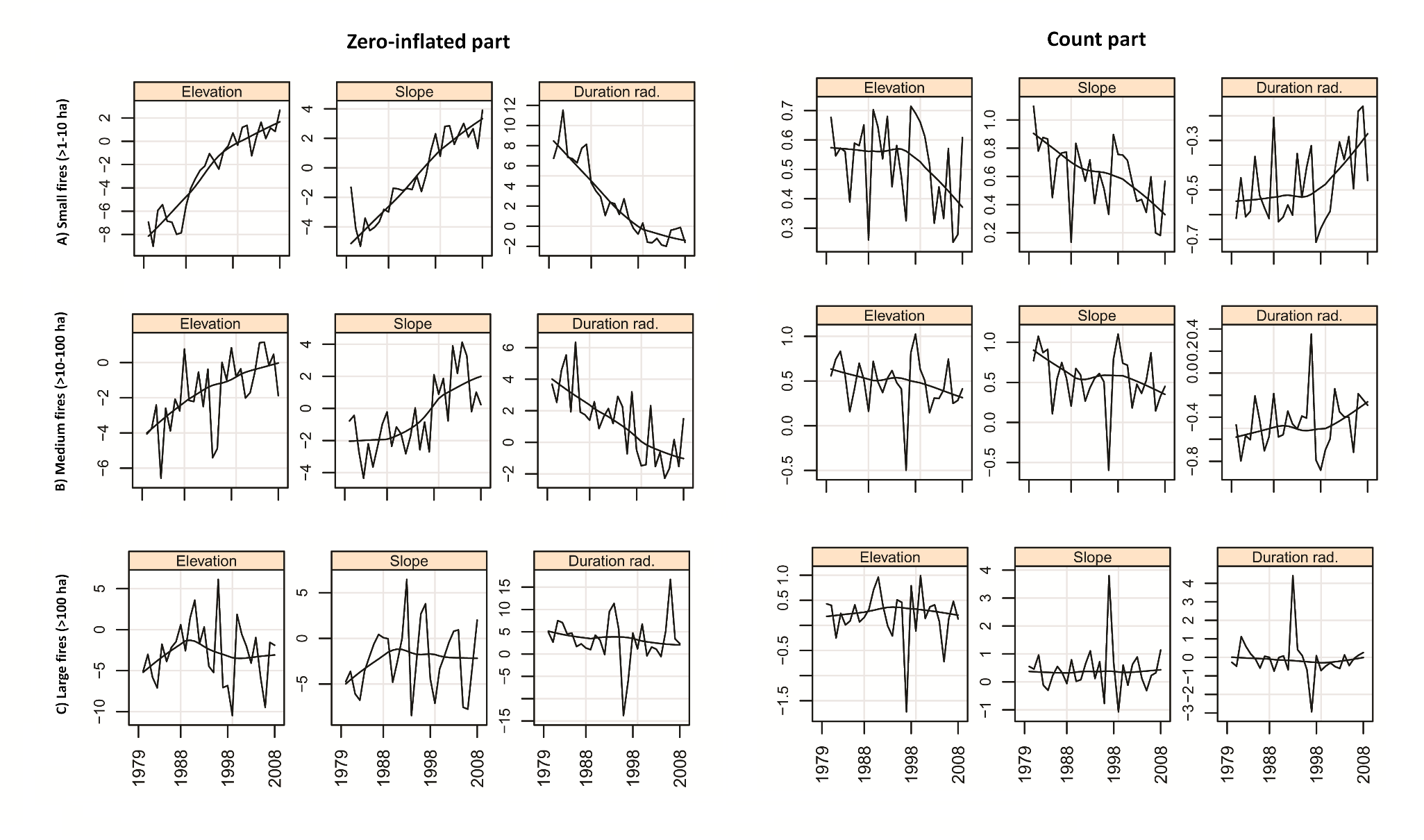


**SM Figure 7.** Temporal dynamic of logit regression coefficients [Ln (X* Time categorical interaction terms)] of fixed co-variates related to topography in the zeros (left panels) and count part (right panels) of univariate longitudinal Zero-Inflated Negative Binomial (ZINB) mixed models for: small fires (≥1 -10 ha) (A), medium fires (>10-100 ha) (B) and large (> 100 ha) fires (C).


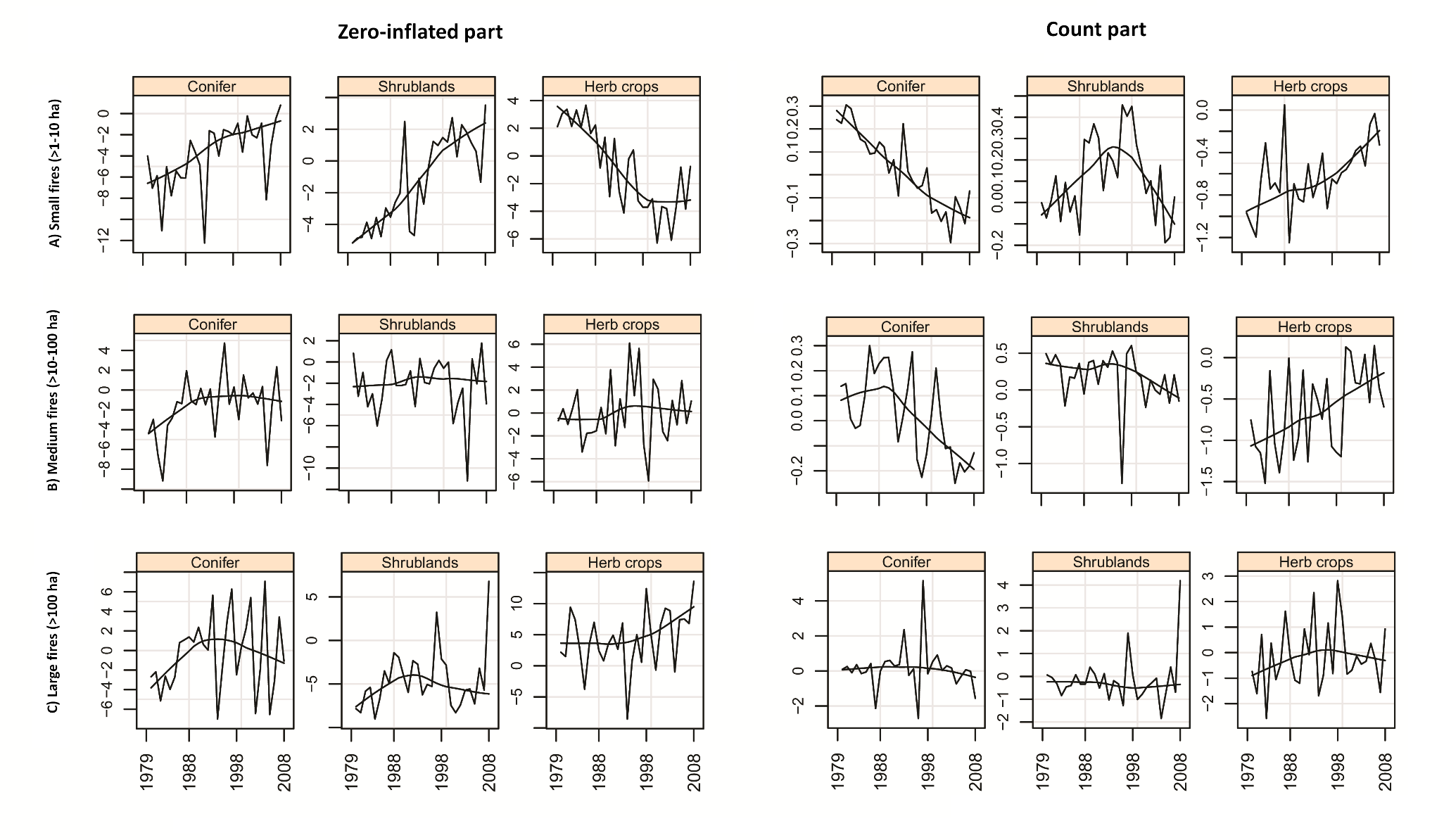


**SM Figure 8.** Temporal dynamic of logit regression coefficients [Ln (X* Time categorical interaction terms)] of fixed co-variates related to Land Use-Land Cover (LULC) types in the zeros (left panels) and count part (right panels) of univariate longitudinal Zero-Inflated Negative Binomial (ZINB) mixed models for small fires (≥1 -10 ha) (A), medium fires (>10-100 ha) (B) and large (> 100 ha) fires (C).

**
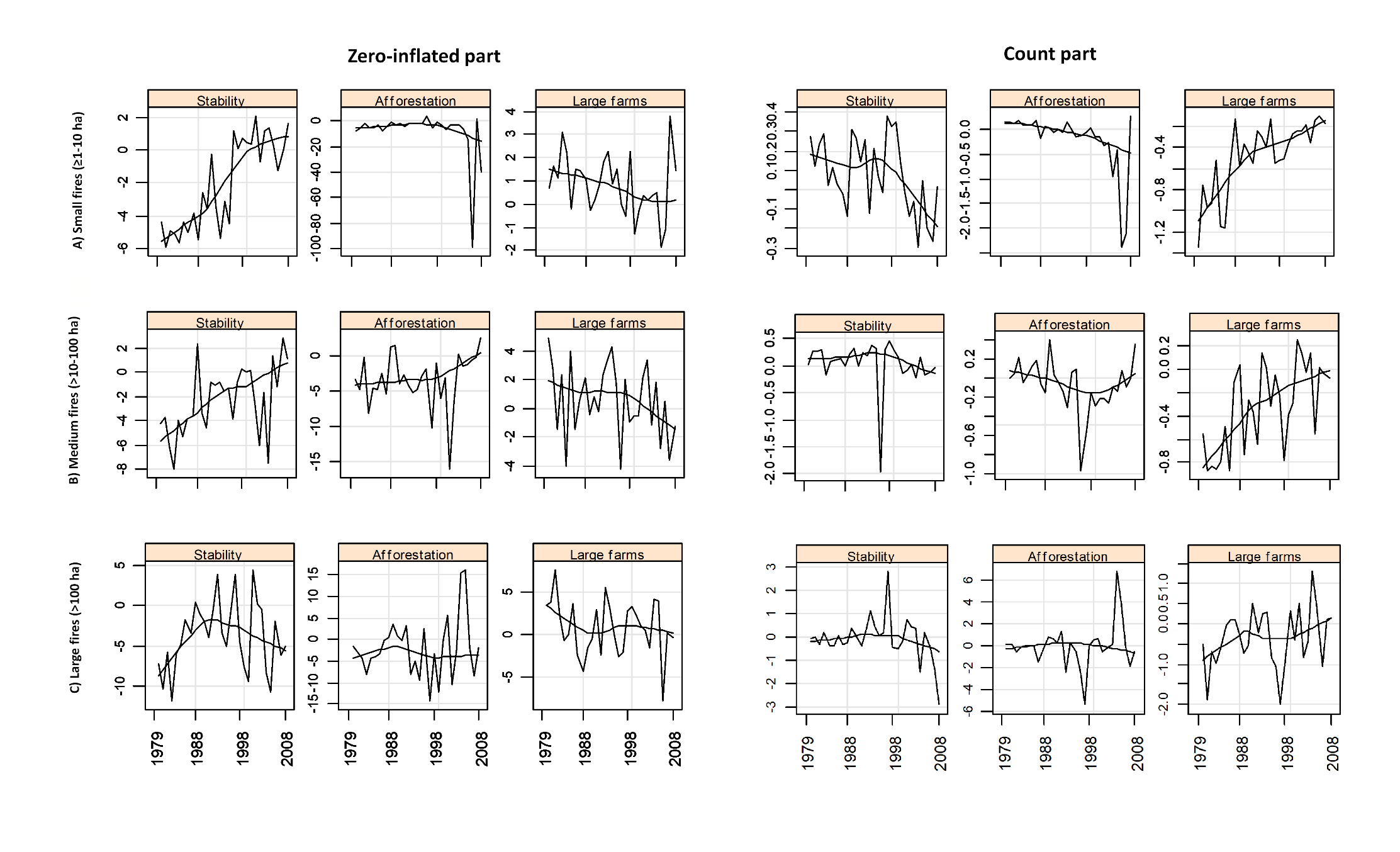
**

**SM Figure 9.** Temporal dynamic of logit regression coefficients [Ln (X* Time categorical interaction terms)] of fixed co-variates related to hazardous stability, farms size (large farms) and population density in the zeros (left panels) and count part (right panels) of univariate longitudinal Zero-Inflated Negative Binomial (ZINB) mixed models for: small fires (≥1 -10 ha) (A), medium fires (>10-100 ha) (B) and large (> 100 ha) fires (C).


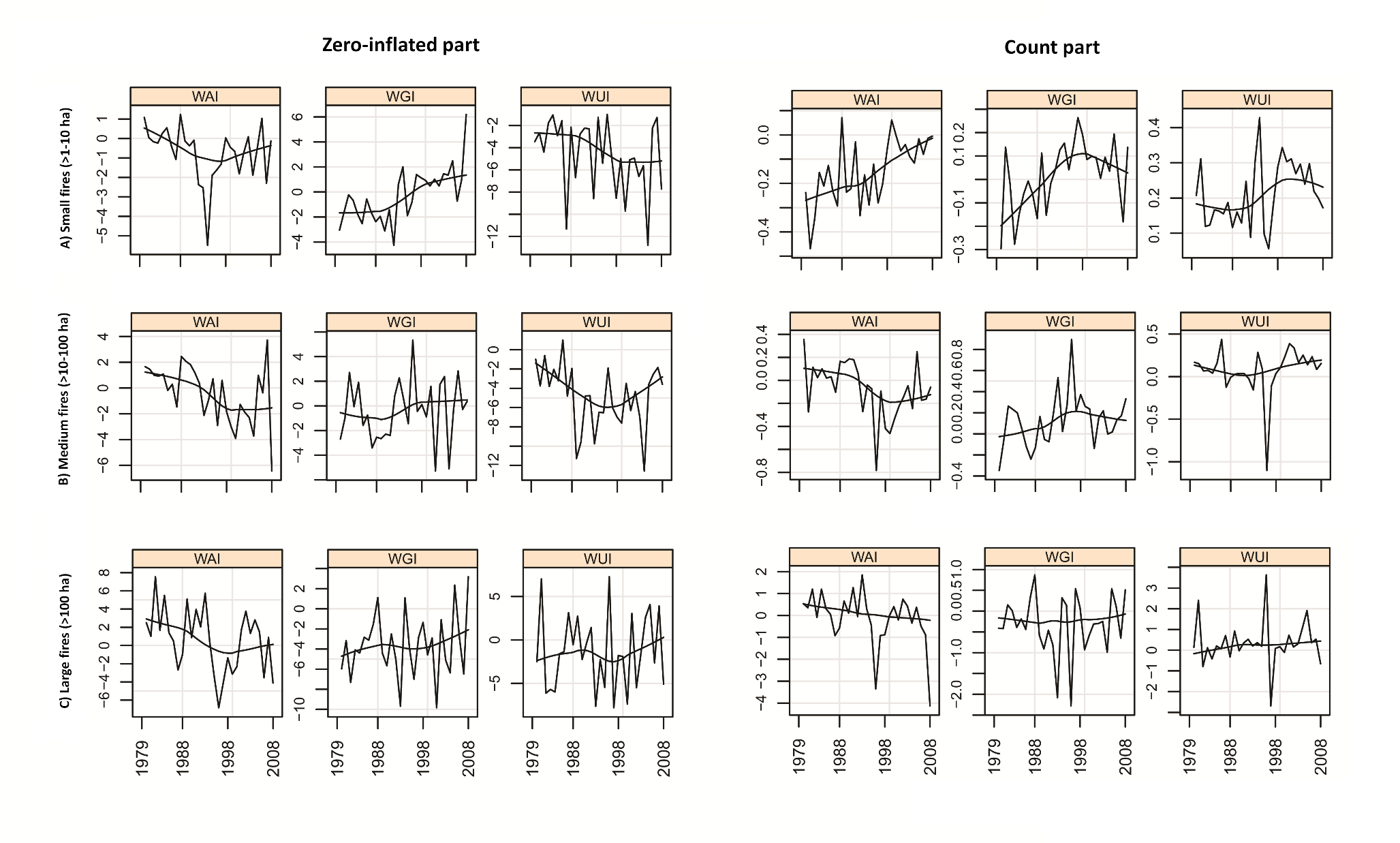


**SM Figure 10.** Temporal dynamic of logit regression coefficients [Ln (X* Time categorical interaction terms)] of fixed co-variates related to forest interfaces (Wildland-Agrarian [WAIs], Wildland-Grasslands [WGIs] and Wildland-Urban [WUIs] interfaces) in the zeros (left panels) and count part (right panels) of univariate longitudinal Zero-Inflated Negative Binomial (ZINB) mixed models for: small fires (≥1 -10 ha) (A), medium fires (>10-100 ha) (B) and large (> 100 ha) fires (C).

# ***References***

Beguería, S., Vicente‐Serrano, S. M., Reig, F. & Latorre, B. Standardized precipitation evapotranspiration index (SPEI) revisited: parameter fitting, evapotranspiration models, tools, datasets and drought monitoring. *International Journal of Climatology* **34**, 3001-3023 (2014).

Bossard, M., Feranec, J., Otahel, J., 2000. CORINE land cover technical guide: Addendum 2000. European Environment Agency Copenhagen.

Herrera, S., Fernández, J. & Gutiérrez, J. Update of the Spain02 gridded observational dataset for EURO‐CORDEX evaluation: assessing the effect of the interpolation methodology. *International Journal of Climatology* **36**, 900-908 (2016).

Martínez, J., Vega-Garcia, C. & Chuvieco, E. Human-caused wildfire risk rating for prevention planning in Spain. *J. Environ. Manage.* **90**, 1241-1252 (2009).

Martínez-Fernández, J., Chuvieco, E. & Koutsias, N. Modelling long-term fire occurrence factors in Spain by accounting for local variations with geographically weighted regression. *Natural Hazards and Earth System Sciences* **13**, 311-327 (2013).

Rodrigues, M., de la Riva, J. & Fotheringham, S. Modeling the spatial variation of the explanatory factors of human-caused wildfires in Spain using geographically weighted logistic regression. *Applied Geography* **48**, 52-63 (2014a).

Rodrigues, M. & de la Riva, J. An insight into machine-learning algorithms to model human-caused wildfire occurrence. *Environ. Model. Software* **57**, 192-201 (2014b).

Vilar del Hoyo, L., Isabel, M. P. M. & Vega, F. J. M. Logistic regression models for human-caused wildfire risk estimation: analysing the effect of the spatial accuracy in fire occurrence data. *European Journal of Forest Research* **130**, 983-996 (2011).

Viedma, O., Moity, N. & Moreno, J. M. Changes in landscape fire-hazard during the second half of the 20th century: Agriculture abandonment and the changing role of driving factors. *Agric., Ecosyst. Environ.* **207**, 126-140 (2015).

Weedon, G. P. et al. The WFDEI meteorological forcing data set: WATCH Forcing Data methodology applied to ERA‐Interim reanalysis data. *Water Resources Research* **50**, 7505-7514 (2014).
